# Beyond Traditional RAFT Polymerization: Emerging Strategies and Future Perspectives; A Third Update

**DOI:** 10.1002/advs.202520657

**Published:** 2026-01-14

**Authors:** Vianna F. Jafari, James L. Grace, Jiajia Li, Joji Tanaka, Glen R. Jones, Athina Anastasaki, Wei You, Jian Zhu, Masami Kamigaito, Cyrille Boyer, Greg G. Qiao

**Affiliations:** ^1^ Polymer Science Group Department of Chemical and Biomolecular Engineering University of Melbourne Parkville Victoria Australia; ^2^ Laboratory of Sustainable Polymers Department of Materials ETH Zürich Zurich Switzerland; ^3^ State and Local Joint Engineering Laboratory for Novel Functional Polymeric Materials Jiangsu Key Laboratory of Advanced Functional Polymer Design and Application College of Chemistry Chemical Engineering and Materials Science Soochow University Suzhou China; ^4^ Department of Chemistry University of North Carolina Chapel Hill North Carolina USA; ^5^ Department of Molecular & Macromolecular Chemistry Graduate School of Engineering Nagoya University Nagoya Japan; ^6^ School of Chemical Engineering The University of New South Wales (UNSW Sydney) Sydney New South Wales Australia; ^7^ Cluster for Advanced Macromolecular Design (CAMD) The University of New South Wales (UNSW Sydney) Sydney New South Wales Australia

**Keywords:** controlled/living polymerization, photochemistry, polymer structures, reversible addition‐fragmentation chain transfer (RAFT)

## Abstract

Reversible addition–fragmentation chain transfer (RAFT) polymerization has undergone transformative growth since its inception in 1998, emerging as a powerful and versatile tool for precision polymer synthesis. This review highlights the latest developments in non‐traditional RAFT polymerization from 2020 to 2025, capturing major innovations in activation techniques and expanding applications. Key emerging directions include the integration of RAFT into smart synthesis platforms powered by artificial intelligence, enabling high‐throughput and autonomous polymer discovery, and innovations in RAFT depolymerization that support sustainable plastic recycling. With RAFT increasingly accessible to diverse materials science domains, this review provides a forward‐looking perspective on the evolving capabilities and future potential of RAFT polymerization.

## Graphical abstract

This review explores recent advances in the past five years for non‐traditional RAFT polymerization, focusing on new activation strategies, sustainable depolymerization, and integration with automated and AI‐driven synthesis. Together, these innovations advance polymer chemistry and reveal how the pieces of the non‐traditional RAFT puzzle are steadily being completed through ongoing development and interdisciplinary progress.

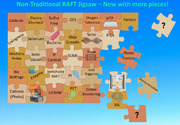

## 1 Introduction

Over the past few decades, there has been significant interest in utilizing reversible deactivation radical polymerization (RDRP) for precision polymer synthesis. Among RDRP techniques, reversible addition‐fragmentation chain transfer (RAFT) developed in 1998 [1] has attracted much attention from the polymer science community owing to its versatility and relative ease of use. The RAFT technique utilizes thiocarbonylthio (TCT) compounds for mediating radical polymerizations, enabling precision polymer synthesis across both industry and academia by regulating chain growth in a degenerative chain transfer process [2, 3, 4]. Over the past two decades, we have observed fast‐tracked progress in the field, rapidly evolving from the traditional thermal activation using radical initiators to diverse activation methods, enabling the use of RAFT in a vast range of reaction conditions and environments to provide better control and versatility, including in biologically friendly environments.

In 2016, we covered this new wave of non‐traditional and alternative activation pathways for the RAFT technique that included direct photoactivation of TCT compounds, as well as the use of a photoredox catalyst for RAFT polymerization. Cationic RAFT polymerization was another interesting and emerging area that was discussed in our 2016 paper, and readers are encouraged to refer to this paper for a more in‐depth discussion on the history of these topics [5].

As the field of RAFT polymerization kept growing, we captured the progress and most recent developments in another highlight article in 2020 [6], discussing the state‐of‐the‐art for non‐traditional RAFT synthesis and novel applications for this technique. Expanding on the topics covered in our 2016 paper, we looked at progress in direct and indirect photoactivation of TCTs, electro‐, sono‐, and cationic photo‐RAFT polymerization techniques. RAFT polymerization in unique environments, including biological settings, and the use of enzymes as activation agents were discussed. Oxygen‐tolerance in RAFT was discussed in detail, and an overview of RAFT polymerization in continuous flow was provided. Emerging applications for the RAFT technique, including polymerization‐induced self‐assembly (PISA), single‐unit monomer insertion (SUMI), synthesis of ultrahigh molecular weight (UHMW) polymers, and the recent developments in automation, combinatorial, and high‐throughput RAFT polymerization, were addressed.

Since 2020, we have observed yet more advances in this ever‐growing field. There have been leaps and bounds on various fronts, from novel sulfur‐free RAFT agents and synthesis techniques to applications in non‐conventional environments, surpassing the traditional thermally activated RAFT polymerization. Such advances are introducing the value of the RAFT technique to a broader audience of materials researchers. Sustainability concerns around “plastics” are being addressed in recent years by new and emerging developments in RAFT depolymerization, enabling efficient recycling of monomer units. Developments in smart and self‐driven synthesis platforms using artificial intelligence (AI) paired with precision RAFT synthesis techniques are helping to automate the fast design of new polymers for different applications and for a better understanding of structure‐property relationships of complex macromolecules. Major developments in oxygen‐tolerant RAFT polymerization have been reported, while RAFT is being performed in unprecedented reaction environments, providing improved reaction kinetics. We therefore deemed it suitable to provide another highlight covering these recent advances. In this article, we explore the latest developments in RAFT polymerization, focusing on significant progress reported from 2020 to 2025. We discuss advancements in novel activation methods and emerging applications, with an outlook on the direction of future research in the field.

## 2 Nontraditional Activation of TCTs

### 2.1 Photoactivation of TCTs

#### 2.1.1 Direct Photoactivation (Photoiniferter)

**FIGURE 1:**
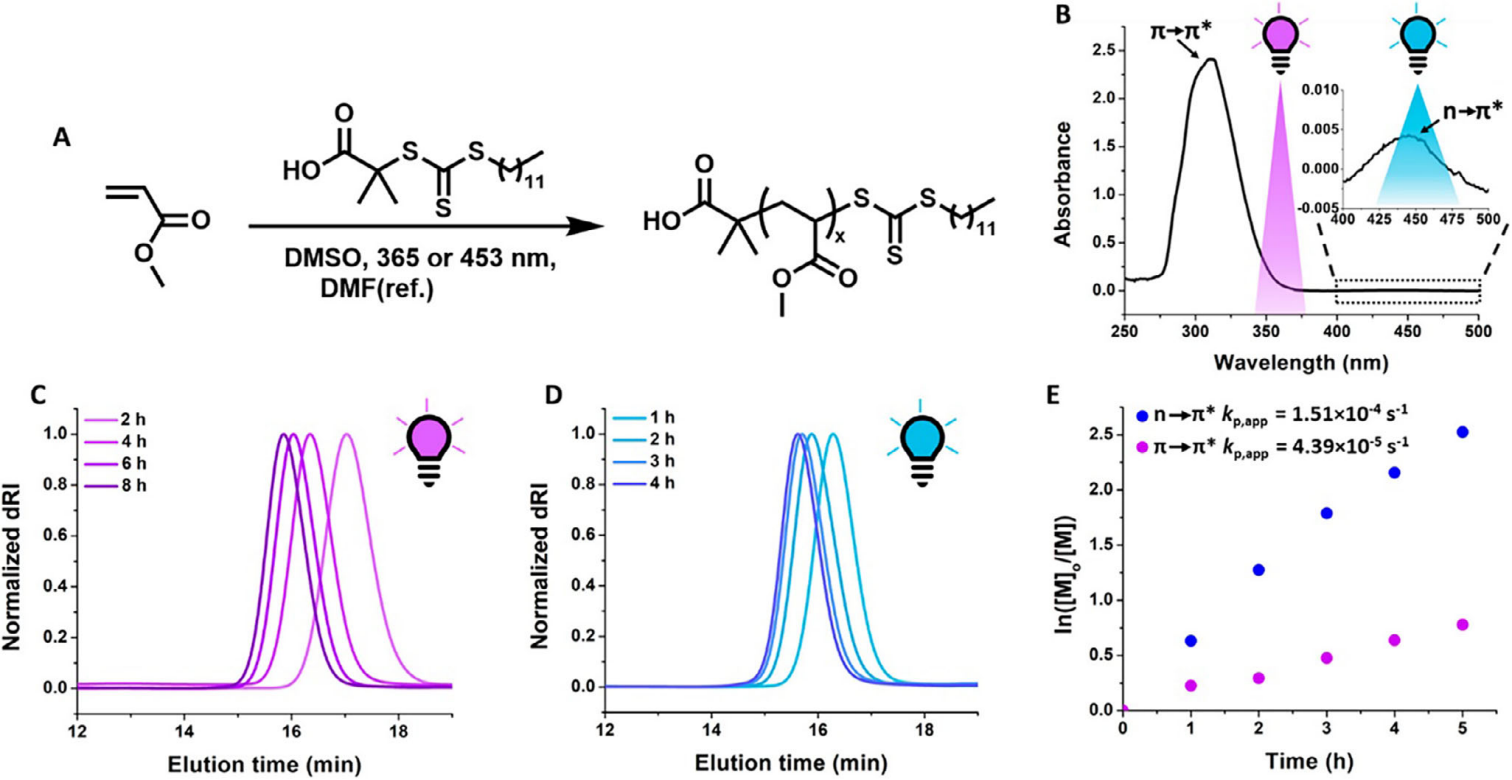
(A) Scheme for the photoiniferter polymerization of methyl acrylate (MA) with 2‐(dodecylthiocarbonothioylthio)‐2‐methylpropionic acid (DDMAT) under different wavelengths of light. (B) UV–vis spectrum of DDMAT in dimethyl sulfoxide, annotated with the location of the electronic excitations and the overlap of the absorbance with the wavelengths of light used as can be seen by the shaded regions. (C) Gel permeation chromatography (GPC) elugrams for the photoiniferter polymerization under 365 nm light. (D) GPC elugrams for the photoiniferter polymerization under 453 nm light. (E) Linear pseudo‐first‐order kinetic plot of the two trithiocarbonate‐mediated photoiniferter polymerizations of MA under different wavelengths of light. Reproduced with permission [17]. Copyright 2022, American Chemical Society.

The seminal work of Otsu [7] in activating thio‐containing compounds using high‐energy photons paved the way for development of methods for the direct activation of RAFT agents. It was later reported independently by the groups of Qiao and Boyer that visible blue light can be used to activate trithiocarbonates or their precursors in what is commonly known as the photoiniferter (PI)‐RAFT technique [8, 9, 10]. In PI‐RAFT, the absorbance of light due to the presence of C═S bond results in the excitation of an electron and a subsequent *β*‐scission of the neighbouring C‐S bond, generating a carbon‐centered R‐group radical in the absence of an exogenous radical source that can be deactivated through reversible combination with the less reactive thiyl radical, also known as the persistent radical [11, 12]. There has been debate over whether the term photoiniferter RAFT polymerization can be used in this case, since RAFT polymerization by definition requires the use of exogenous radicals, and thiocarbonylthio compounds participate in degenerative chain transfer. Photoiniferter polymerization has been suggested as a more technically accurate term to describe the phenomenon in the direct photo‐activation of RAFT agents. Nonetheless, the absence of an exogenous radical source significantly improves chain‐end fidelity [13], which has resulted in the synthesis of ultrahigh molecular weight polymers in recent years [14, 15, 16]. It is well understood that the selection of UV versus visible light for activation of RAFT agents targets different electronic excitations of the iniferter molecule (Figure 1) [17]. The energy gap for the π → π* and the n → π* electronic transitions of the C─S bond homolysis in the iniferter molecule are different, resulting in a n → π* transition at longer wavelengths. Selection of wavelength to target different electronic excitations of the iniferter can result in different levels of control on reaction rate and molecular weight control. It was shown that both trithiocarbonates and xanthates exhibit a faster polymerization rate due to the higher quantum yield of the n → π* transition, resulting in a higher radical concentration.

PI‐RAFT offers facile polymerizations with spatiotemporal control, while the livingness of chains can be enhanced due to the absence of an exogenous radical source. However, slow reaction speeds can hinder its wider application. Increasing light intensity has been considered as a straightforward strategy to increase radical flux to get faster polymerizations [18, 19, 20], although usually with a loss of livingness and/or control on polymerization, partly due to the increase in reaction temperature when exposed to intense light. Recent findings on the design and use of xanthate RAFT agents with more electron‐donating Z groups have resulted in an enhanced photodissociation of the RAFT agent, improving the reaction speeds to an unprecedented minute‐scale for aqueous systems under visible light, giving an advantage over UV‐mediated xanthate‐based systems [21, 22]. To enable fast polymerization under low intensities of visible light, alternative solvents, including ionic liquids (ILs) and deep eutectic solvents (DESs) have been explored in recent years, which have shown rate enhancement effects as well as oxygen tolerance. Qiao's group have reported the use of ILs as reaction solvent in organic and aqueous PI‐RAFT systems under visible light with a significant increase in reaction speed without compromising livingness or control of MW and dispersity [23, 24]. A similar observation was reported by Yu and coworkers [25] with the use of DESs as solvent in the iniferter RAFT polymerization of acrylamides, (meth)acrylates, and styrene. While polarity effects and enhanced photo‐stability of RAFT agents are thought to play a role in this observation, a more in‐depth study of these solvents is required to understand the effect of ILs and DES on oxygen tolerance and rate enhancement effects.

#### 2.1.2 Indirect Photoactivation (PET‐RAFT)

**SCHEME 1:**
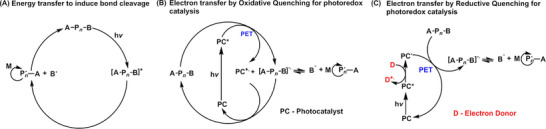
Photomediated controlled/radical polymerization (photo‐CRP). (A) PI‐RAFT polymerization; PET‐RAFT polymerization: (B) photoredox‐catalyzed electron transfer through an oxidative cycle, and (C) photoredox‐catalyzed electron transfer through a reductive cycle. Adapted with permission [41]. Copyright 2019, John Wiley and Sons.

Photoinduced electron/energy transfer–reversible addition fragmentation chain transfer (PET‐RAFT) polymerization was first reported by Boyer's group in 2014, utilizing tris[2‐phenylpyridinato‐C2,N]iridium(III) (Ir(ppy)_3_) and tris(bipyridine)ruthenium(II) chloride (Ru(bpy)_3_Cl_2_) as photocatalysts (PCs) activated under low‐intensity blue light. The use of PCs to drive PET‐RAFT polymerization follows a photoiniferter (PI) mechanism, wherein radicals are formed from the R‐group of the RAFT agent (Scheme 1) [9, 26]. However, in PET‐RAFT, the processes of light absorption (by the PC) and activation (of the RAFT agent) are decoupled. This decoupling enables PCs, which possess superior photophysical characteristics compared to RAFT agents, to achieve more efficient RAFT activation and better control over polymerization [27]. Furthermore, the ability to operate across a broader range of wavelengths provides milder reaction conditions and allows the incorporation of latent chromophores that would otherwise be prematurely activated at higher‐energy wavelengths [28, 29]. Therefore, substantial efforts have focused on discovering new PCs capable of activation under longer wavelengths, including green, red, far‐red, and near‐infrared (NIR) light, as well as enhancing polymerization kinetics. In the quest for PCs activated under low‐energy wavelengths, chlorophyll, Zn‐porphyrin, bacteriochlorophyll, and a persistent radical anion of perylene dianhydride have been reported for use under visible and near‐infrared/far‐red light [30, 31, 32, 33]. More recently, a diverse range of PCs has been developed to enable PET‐RAFT polymerization under NIR light, facilitating polymerization in opaque systems, overcoming a major limitation of visible light‐activated polymerization requiring the use of transparent reactors and reaction mixtures [34]. For example, zinc phthalocyanine, zinc phthalocyanine covalent polymers, hollow conjugated microporous photocatalysts, aluminum phthalocyanine, and aluminum naphthalocyanine have demonstrated excellent performance in activating PET‐RAFT under aerobic conditions, often with the assistance of cocatalysts (e.g., tertiary amines) or peroxide additives [35, 36, 37, 38, 39]. These advancements enable polymerization through visible light‐opaque barriers and improve performance in non‐homogeneous environments, such as dispersed polymerization systems [36]. For instance, using this photoactivated system, Boyer's group demonstrated polymerization‐induced self‐assembly (PISA) of RAFT‐terminated polyethylene glycol with hydroxypropyl methacrylamide (HPMA) as the core‐forming block, employing zinc phthalocyanine as the photocatalyst [36]. This work successfully produced a range of morphologies, including spherical micelles, cylindrical micelles, and vesicles. More importantly, the polymerization kinetics was not affected by the change in transparency of the PISA reaction. Expanding the range of wavelengths to operate PET‐RAFT has also opened the possibility of using different colors of light (or wavelengths) to drive distinct chemical reactions [40], such as polymerization and dimerization, enabling the precise control the formation of crosslinked nanoparticles [29].

In addition to broadening the range of light wavelengths suitable for PET‐RAFT polymerization, significant progress has been made in developing heterogeneous photocatalysts for rapid catalyst recovery [42]. Metal‐organic frameworks (MOFs) [43, 44], covalent organic frameworks (COFs) [45, 46], fibers [47], and carbon and metallic nanoparticles [48, 49, 50] have been successfully employed. These heterogeneous systems enable efficient catalyst recovery and removal from the resulting polymers, enhancing the sustainability and practicality of the process.

Another critical research direction has been the development of faster PET‐RAFT polymerization through the incorporation of reducing agents, resulting in the reductive PET‐RAFT process [51] and the design of new PCs [52, 53, 54]. For example, adding tertiary amines accelerates the reaction by donating electrons to the photocatalyst, which subsequently transfers them to the RAFT agent and promotes its dissociation. Much of this work has utilized xanthene compounds such as eosin and erythrosine [54]. Under reductive conditions, PET‐RAFT polymerization achieves significantly enhanced kinetics. For instance, adding triethanolamine (TEOA) to PET‐RAFT activated by erythrosine enables over 95% monomer conversion in under 10 min for dimethyl acrylamide (DMA), compared to over 60 min required in the absence of TEOA to reach a similar conversion [55]. Although the reductive PET‐RAFT process offers significant improvements in reaction speed compared to conventional oxidative PET‐RAFT, it is associated with higher dispersity values (typically around 1.2–1.3 for reductive PET‐RAFT versus less than 1.1 for the oxidative process with the same monomer). This increased dispersity is attributed to the higher radical concentrations inherent to the rapid kinetics of the reductive process.

### 2.2 Electro‐RAFT

**SCHEME 2:**
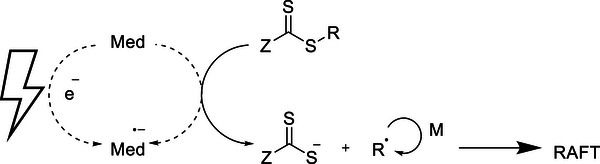
Electrochemically mediated RAFT (eRAFT) polymerization.

An alternative for externally regulated RAFT polymerization is electrochemical RAFT (electro‐RAFT or eRAFT). Electro‐RAFT works by the generation of radicals via the electroreduction under an applied electrical field of either the RAFT agent or exogenous initiator, either directly or using a mediator (Scheme 2). In the past couple of years, there have been a number of papers on the grafting of ferrocenyl polymers onto an electrode surface through eRAFT polymerization as a biosensor and extensively investigated for their ability in sensing nucleic acids [56], matrix metalloproteinase [57], trypsin [58], and aflatoxin [59] at ultralow concentrations.

The Moad group has also investigated the combination of emulsion polymerization and eRAFT, to overcome what they called limitations of effective eRAFT, including the passivation by radical species and various side reactions involving the RAFT agent at the working electrode. As such, they reported eRAFT that was conducted in emulsion allowed for electrolytes and mediators required to be located only in the aqueous continuous phase, separate from the precursor amphiphilic macroRAFT agent and the forming macroRAFT agent product that is in the dispersed or particle phase [60, 61]. By using a “conventional” iron‐persulfate redox couple, a rapid eRAFT polymerization was able to be achieved with high conversion, low dispersity for a multiblock copolymer system, and could be temporally controlled [60].

### 2.3 Sono‐RAFT

**SCHEME 3:**
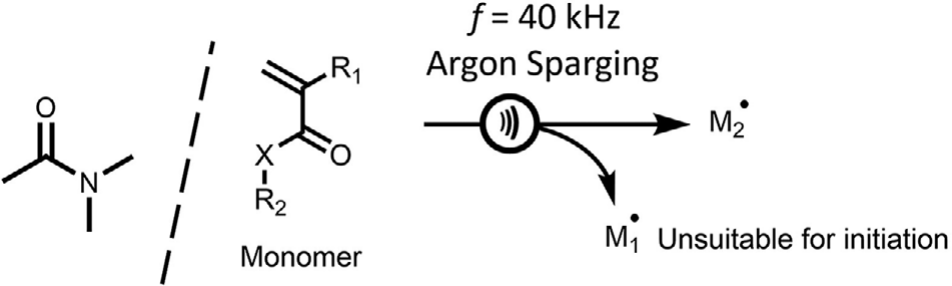
Sonochemical formation of radical initiators via low‐frequency ultrasound (f = 40 kHz) in bulk or DMAc, M_1_• represents the complementary radical to M_2_^•^ that does not participate in the initiation of polymerization. Adapted with permission [63]. Copyright 2023, American Chemical Society.

Sono‐RAFT relies on the activation of the TCT group through the direct generation of radicals via the sonication of the reaction solvent. Sono‐RAFT, when first reported by Qiao and coworkers used high‐frequency (414 and 490 kHz) ultrasound to cause acoustic cavitation of the solvent to generate radicals [62]. Recent work by Sumerlin and co‐workers were able to employ low frequency ultrasound (40 kHz) to generate initiating radical species (Scheme 3). It was found that the continuing introduction of an inert gas during sonication was essential to promote the generation of the initiating radical species, allowing for well‐controlled living polymers with narrow dispersity (Ð = 1.04) [63].

**FIGURE 2:**
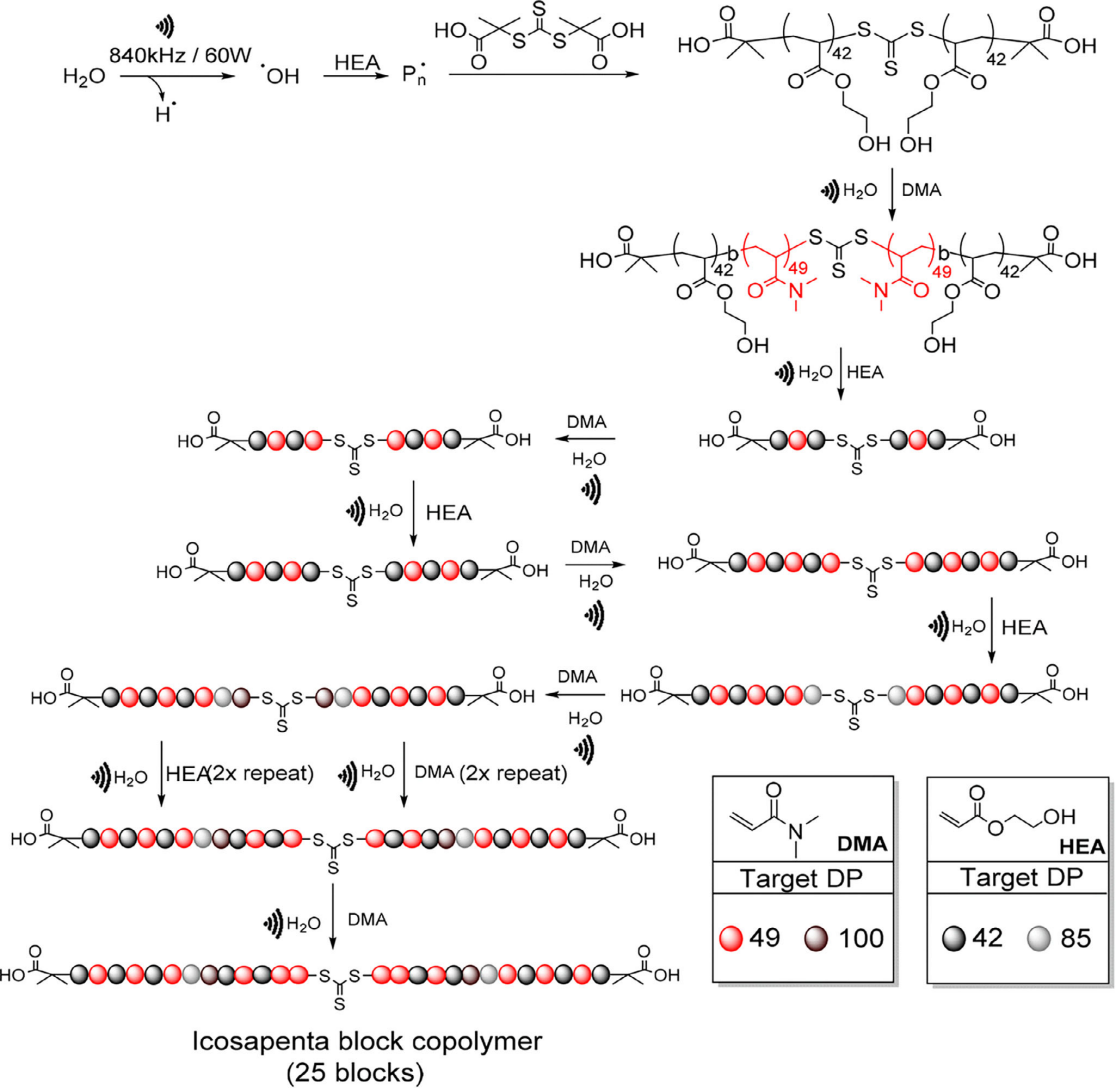
Schematic representation of the synthesis of icosapenta block copolymer of HEA and DMA via sono‐RAFT. Reproduced with permission [65]. Copyright 2022, Royal Society of Chemistry.

Recently, the Qiao group combined sono‐RAFT with a continuous flow method and reported significant advantages to this process most notably that it allowed for higher monomer concentration than conventional batch reactions. Additionally, it was found that the tubing material used in the flow system had an effect on the polymerization, with the stainless steel microreactors resulting in increased cavitational intensity and decreased oxygen contamination compared to PFA tubing [64]. The same group also showed the high chain‐end fidelity of sono‐RAFT through multiple chain extensions, being able to synthesize a 25 block copolymer within 46 h at room temperature, which showed controlled molecular weight with a final dispersity of 1.39 (Figure 2) [65].

Furthermore, the advancement of ultrasonic polymerization in the future is expected to involve the integration of real‐time monitoring and automation. These innovations aim to improve continuous polymerization control, efficiency, and sustainability even more. In addition, the investigation of new monomers, the effect of reactor surface area, applied power, and frequency can result in the development of novel polymerization techniques with enhanced properties and a broader range of applications.

### 2.4 Cationic (Photo‐)RAFT

**FIGURE 3:**
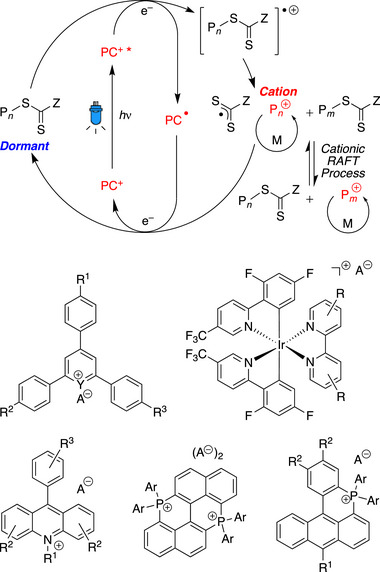
Cationic RAFT polymerization using photoredox catalysts.

Since the pioneering works on the cationic RAFT polymerization of vinyl ethers using TCT with triflic acid in 2015 [66] and photoredox catalysts in 2016 [67], cationic RAFT or degenerative chain transfer (DT) polymerization has also been widely developed in terms of catalysts, stereochemical control, monomer scope, combination with other polymerizations, continuous flow systems, 3D printing, and SUMI [68, 69, 70]. In addition to pyrylium and iridium photoredox catalysts, other oxidizing catalysts, such as phosphonium and acridinium salts (Figure 3), have been proven by the Fors, Liao, and Kamigaito groups to be effective for the cationic RAFT polymerization of vinyl ethers in conjunction with trithiocarbonates, dithiocarbamates, or thioacetals [71, 72, 73, 74, 75, 76, 77]. Cationic RAFT polymerization can also occur under mild conditions with the use of a strong organic acid and a hydrogen bond donor [78]. Zhu and coworkers reported that homogenous and heterogeneous selenonium salts function as metal‐free Lewis acid catalysts [79, 80], and this same group also reported radical‐promoted cationic RAFT polymerization using manganese, iron, iridium, and zinc catalysts under light in the visible to near‐infrared region [81, 82, 83]. Additionally, the scalable synthesis of a solid and easily isolable cationic RAFT agent was reported by Fors and coworkers [84].

One of the features distinguishing cationic polymerization from radical polymerization is that the stereochemistry of polymer chains can be controlled by the counteranion. Stereoselective cationic RAFT polymerization of vinyl ethers was demonstrated by the groups of Leibfarth [85] and Liao [86, 87, 88] by the use of chiral BINOL‐derived phosphoramide‐type Brønsted acid catalysts to produce isotactic poly(vinyl ethers) with controlled molecular weights and stereoblock polymers. Cationic RAFT polymerization of bulky vinyl ethers such as trimethylsilyl vinyl ether yields an isotactic‐rich polymer, which can be further used as a macro RAFT agent for the radical RAFT polymerization of vinyl acetate, finally generating a stereoblock poly(vinyl alcohol) after deprotection of the silyl and acetyl groups [89]. Uchiyama et al. also reported the asymmetric cationic DT polymerization of benzofuran using chiral β‐amino acid additives to produce optically active polymers with controlled molecular weights [90].

Although *p*‐alkoxystyrene can be polymerized in a controlled manner using alcohol and phosphate as the DT or RAFT agent [91, 92], controlled cationic RAFT polymerization of unsubstituted styrene is still challenging [93]. α‐Methylstyrene and isobutylene, both of which are 1,1‐disubstituted monomers, can be polymerized in a controlled manner using *exo*‐olefins as sulfur‐free RAFT agents, wherein the C═C bond undergoes cationic addition followed by fragmentation via the β‐scission of the cationic intermediate [94]. Cyclic monomers such as oxiranes [95], cyclic enol ethers [96], glucose 1,2,4‐orthopivalates [97], and cyclic thioacetals [98, 99] also undergo ring‐opening cationic RAFT or DT polymerization in the presence of appropriate chain‐transfer agents to produce unique polymers that are distinct from vinyl polymers. In particular, main‐chain thioacetal bonds that form via the ring opening of a cyclic thioacetal function as in‐chain dormant bonds for the subsequent cationic DT polymerization of vinyl ethers to produce poly(vinyl ethers) with degradable thioacetal bonds in the main chains.

Combinations of cationic RAFT polymerization and other polymerizations can yield a variety of unique copolymers that cannot be obtained with a single polymerization methodology. There have been reports of interconvertible radical and cationic polymerizations using photo‐ and electrochemical stimuli, which generate cationic and radical propagating species, respectively, from the trithiocarbonate bonds as common RAFT terminal to produce vinyl ether and acrylate copolymers with unique monomer sequences [100, 101]. Ring‐opening polymerization [102, 103] and step‐growth polyaddition [104, 105] have also been combined with cationic RAFT polymerization to produce unique block and multiblock copolymers.

Cationic RAFT polymerization has also been employed in continuous flow systems [106, 107] and for dispersity control [108, 109, 110], thermosets [111], gels [112], 3D printing [113, 114, 115, 116, 117], and SUMI [118, 119] applications, as in the radical RAFT polymerization described below, although the details are omitted here.

### 2.5 Fenton ‐and Photo‐Fenton‐ RAFT

The use of Fenton chemistry in RAFT polymerization was first reported in 2017 [120] by Qiao's group, where fast polymerizations of acrylates and acrylamides in aqueous systems was achieved within minutes at room temperature. The catalytic decomposition of hydrogen peroxide in the presence of ferrous ions resulted in the production of a high flux of hydroxyl radicals. Since then, Fenton chemistry for RAFT polymerization has been explored to accommodate different reaction environments such as bio Fenton‐RAFT where hemoglobin in red blood cells was used as an iron source for Fenton activation which paves the way for more advanced cell‐polymer applications [121]. The addition of glucose oxidase (GOx) enzyme to the reaction for the enzymatic production of hydrogen peroxide was another pivotal development in Fenton‐mediated RAFT polymerization. GOx utilizes molecular oxygen to produce hydrogen peroxide and so can play a dual role of degassing‐agent and an initiating catalyst. This has led to developments such as the synthesis of ultra‐high molecular weight polymers, taking advantage of the slow and gradual release of initiating radicals when GOx is present [122], and the automated synthesis of high‐order multiblock copolymers with excellent chain‐end fidelity [123]. Fenton chemistry for RAFT polymerization, however, has some inherent limitations, including lack of temporal control and catalyst contamination. Iron contamination for biomedical applications has been addressed by the development of iron‐containing metal‐organic frameworks (MOF) as heterogeneous catalysts for aqueous systems [124], as well as magnetic MOF nanoparticles that also enable temporal control using a magnet to remove the heterogeneous catalyst [125]. Recently, Qiao's group [126] reported the expansion of Fenton chemistry to organic media for the synthesis of hydrophobic acrylate and methacrylate class monomers using MOF nanoparticles. To enhance enzyme stability and combat their susceptibility to deactivation, bionic enzymes have also been reported. Bionic enzymes such as covalent organic frameworks (COF) are produced as heterogeneous, chemically stable catalysts that possess enzymatic properties and can produce active species for radical polymerization. For example, photobionic COFs have been reported to utilize oxygen and water with the help of an electron sacrificial agent such as TEA to generate hydroxyl radicals at ambient temperature under visible light [127].

**FIGURE 4:**
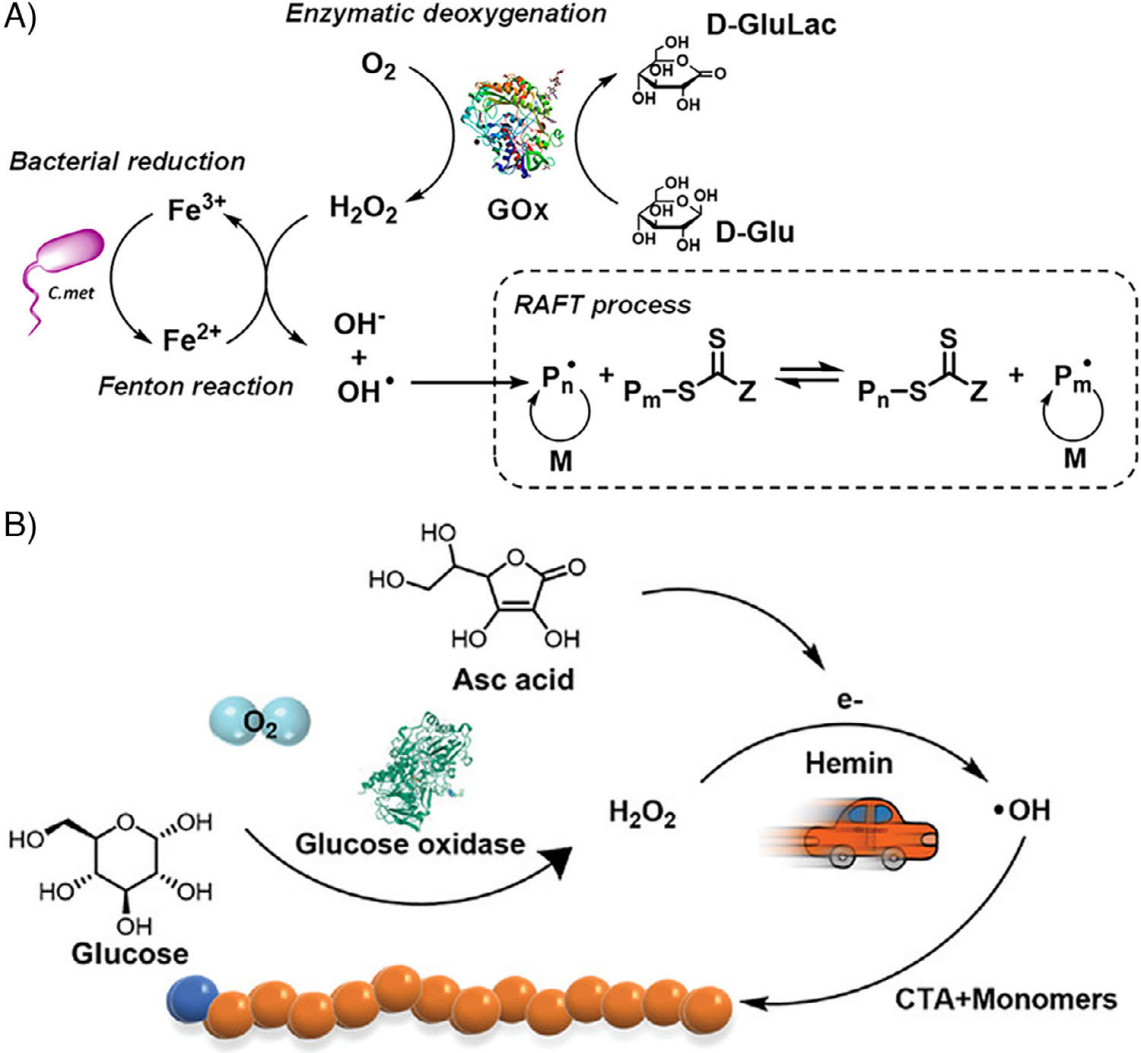
A) Fenton GOx RAFT Process initiated by bacteria. Reproduced with permission [128]. Copyright 2022, American Chemical Society. B) Fenton GOx RAFT Process Initiated by ascorbic acid and hemin. Reproduced with permission [129]. Copyright 2022, John Wiley and Sons.

Alternative strategies have been explored to enhance reaction rates for Fenton chemistry. In 2022 [128] a modified Fenton reaction was reported; this aqueous oxygen‐tolerant system is initiated by Cupriavidus metallidurans CH34 bacteria which has iron reducing capabilities. The bacteria can reduce Fe^3+^ to form Fe^2+^ in situ to enhance reaction rates, with GOx for degassing and the production of hydrogen peroxide (Figure 4A). In another recent work [129], a so‐called bio‐enhanced RAFT polymerization using GOx, glucose, ascorbic acid, and hemin is reported, where hydrogen peroxide is generated by GOx, glucose, and oxygen, and is then activated by hemin and reacted with ascorbic acid to generate hydroxyl radicals (Figure 4B).

Recently, photo Fenton‐like RAFT has been reported as a means to improve temporal control in the production of the very reactive hydroxyl radicals. In 2024 [130] ferrocene‐directed photo‐Fenton RAFT polymerization was reported, where the focus was on organometallic compounds such as ferrocenyl derivatives with modified functionalities which can generate hydroxyl radicals through photo‐promoted Fenton chemistry upon irradiation by visible blue light to generate hydroxyl radicals to produce polymers with different end‐groups, facilitating their self‐assembly into hierarchical architectures. Methylene blue has also been reported as a photo sensitizer for the activation through photolysis of hydrogen peroxide via a photo Fenton‐like reaction for the production of hydroxyl radicals under red light, reported both for aqueous and organic solvent systems [131, 132].

### 2.6 Mechanoredox RAFT

**FIGURE 5:**
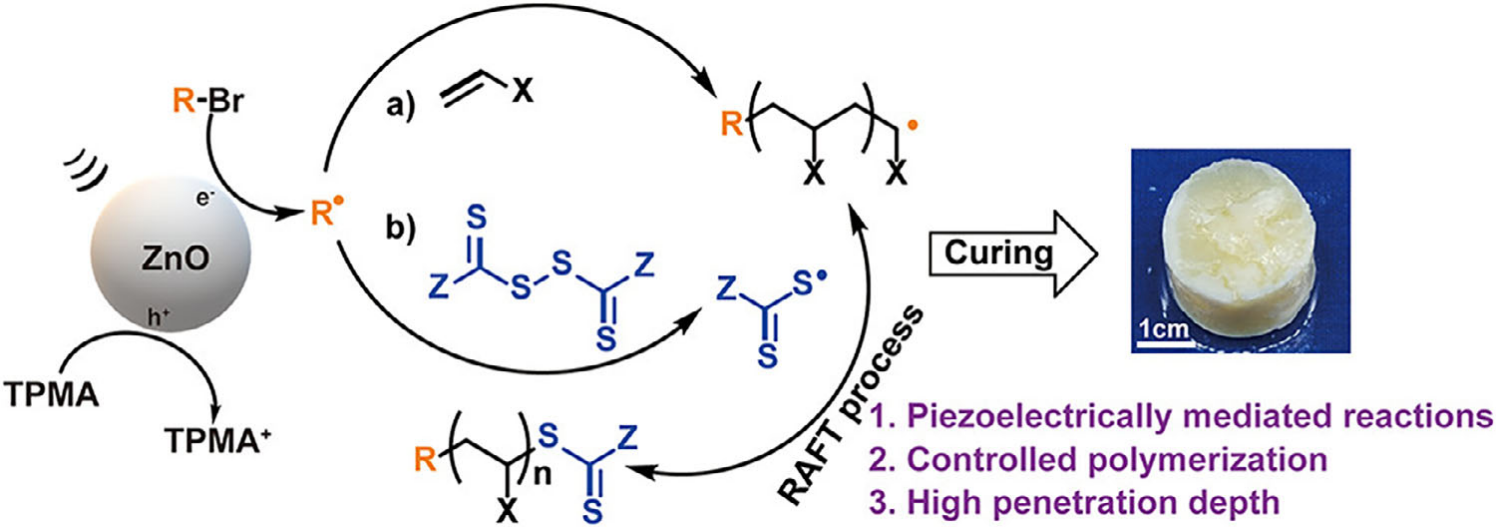
Mechanoredox‐catalyzed RAFT polymerization by ultrasound using ZnO as piezoelectric materials. Reproduced with permission [137]. Copyright 2022, American Chemical Society.

Mechanoredox catalysis offers an alternative approach for activating TCTs. This method utilizes piezoelectric materials subjected to external mechanical stimuli, which is able to initiate redox reactions through single electron transfer [133, 134, 135, 136]. Wang et al. reported the first mechanoredox RAFT polymerization (Figure 5) [137], employing zinc oxide (ZnO) nanoparticles as the piezoelectric materials to cleave alkyl bromide under ultrasound, thereby initiating polymerization and triggering the disulfide bond of the bis(trithiocarbonate) bisulfide to generate RAFT agent in situ. While the polymerization of (meth)acrylates exhibited a relatively narrow molecular weight distribution (*Ð* < 1.40), the experimental molecular weights of these polymers were significantly higher than the theoretical values, attributed to the low efficiency of the piezoelectric‐mediated process and subsequent CTA activation. To enhance the efficiency of the piezoelectric materials, the same group screened various ZnO crystals with different polar facets ratios prepared from different zinc precursors (Zn(NO_3_)_2_, ZnSO_4_, ZnAc_2_, and ZnCl_2_) [138]. A near‐quantitative initiator efficiency was achieved using ZnO prepared by Zn(NO_3_)_2_, and the experimental molecular weights of the polymers closely matched the theoretical values.

**FIGURE 6:**
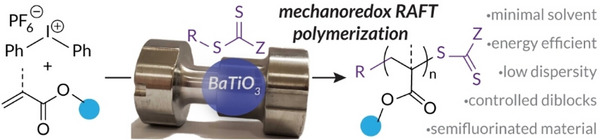
Mechanoredox‐catalyzed RAFT polymerization by ball milling using BaTiO_3_ as piezoelectric materials. Reproduced with permission [140]. Copyright 2022, John Wiley and Sons.

Compared to conventional solution‐state systems, ball milling is more energy‐efficient and requires only minimal amounts of solvent [139]. Golder's group employed BaTiO_3_ as the piezoelectric materials to reduce diphenyl iodonium hexafluorophosphate (DPIHP), generating initiating radicals for RAFT polymerization (Figure 6) [140]. This approach successfully produced well‐defined poly(meth)acrylates, telechelic polymers and a semifluorinated diblock, showcasing its versatility.

**FIGURE 7:**
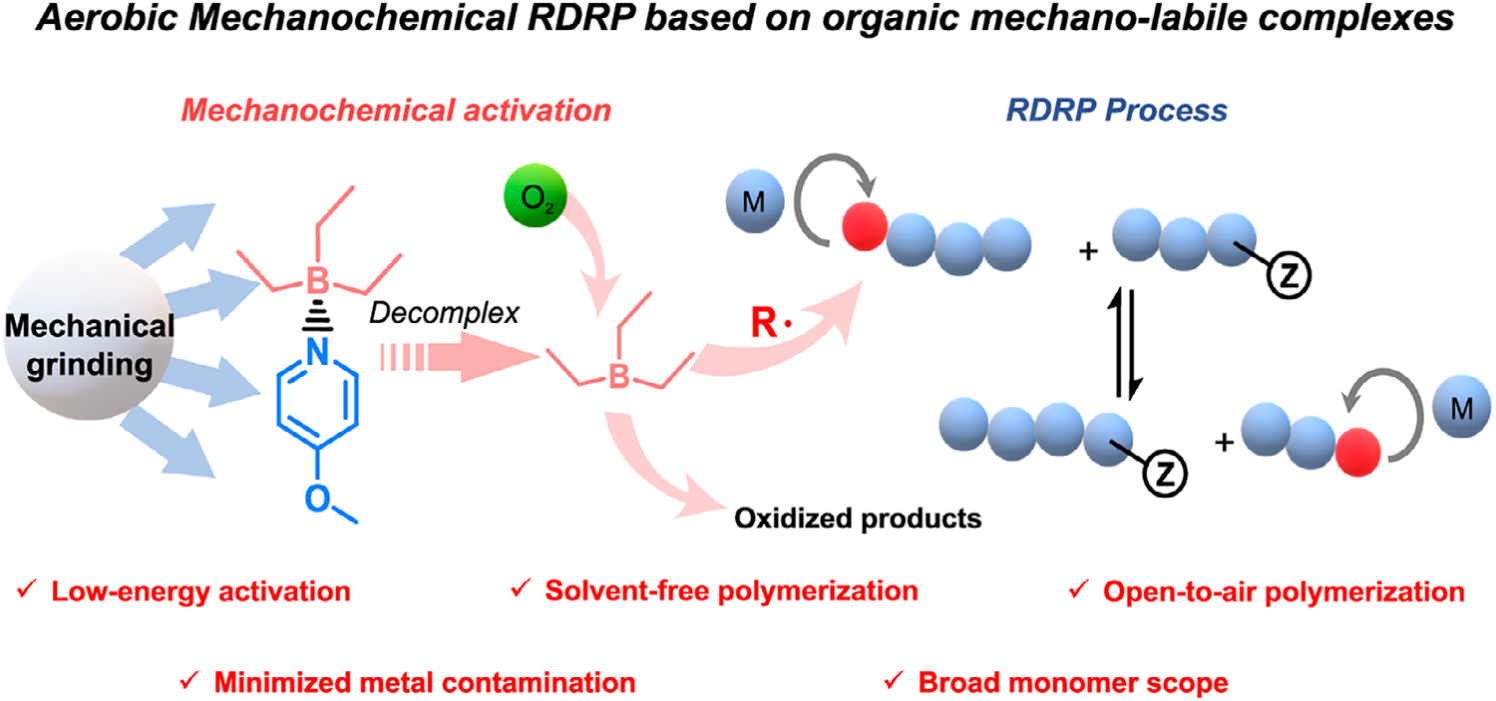
Aerobic Mechano‐RAFT polymerization. Reproduced with permission [141], Copyright 2024, Springer Nature.

In another recent study, an Et_3_B/pyridine complex was utilized as a mechano‐labile initiator, releasing Et_3_B upon ball milling [141]. This released Et_3_B can subsequently react with molecular oxygen to generate initiating radicals for RAFT polymerization (Figure 7). This aerobic system facilitates not only the controlled polymerization of various vinyl monomers but also the synthesis of polymer/perovskites hybrids without solvents or degassing.

### 2.7 Acid‐Triggered RAFT Polymerization

In 2024 Anastasaki and colleagues reported that acids were capable of triggering and accelerating RAFT polymerizations in the absence of a conventional radical source (i.e. a radical initiator) [142]. Initial experiments showed that heating an aqueous solution of *N,N*‐dimethyl acrylamide (DMAm) with a trithiocarbonate RAFT agent at 70°C, without any external radical initiator, resulted in no polymerization. However, quantitative monomer conversion could be achieved by simply lowering the solution's pH with small amounts of sulfuric acid. Notably, higher polymerization rates were observed at lower pH values. Gel permeation chromatography (GPC) revealed narrow, monomodal molar mass distributions (*Ð* = 1.12–1.14), indicating successful RAFT polymerizations when acid was present. Additionally, excellent end‐group fidelity was demonstrated through chain‐extension experiments and confirmed by MALDI‐ToF‐MS. A key advantage of acid‐triggered RAFT polymerization is the apparent suppression of termination events, which was demonstrated by comparing the one‐pot synthesis of triblock copolymers under optimized conditions with either conventional initiation or acid‐triggered initiation. The acid‐triggered system yielded a triblock with a final dispersity of 1.16 with noticeably less tailing to low molar mass observed in the GPC chromatograms than conventional initiation (*Ð* = 1.28). The scope of acid‐triggered RAFT polymerization encompasses a range of monomer classes, solvents, and RAFT agents, with both strong and weak acids (e.g., citric acid) capable of triggering polymerization.

The mechanism of radical generation in the presence of acid was investigated through control reactions conducted without the chain transfer agent (CTA). These experiments revealed that the CTA does not participate in the initiation process. Instead, a Flory‐type autoinitiation [143] mechanism between two monomers was proposed, leading to the formation of a diradical species. This hypothesis was supported by density functional theory (DFT) calculations, which showed that protonation of the monomer significantly lowers the activation barrier for autoinitiation, increasing the rate of radical formation by 3.5 orders of magnitude compared to non‐protonated monomers. Additionally, protonation of the monomer was calculated to enhance the propagation step, with rate coefficients approximately an order of magnitude higher than in non‐protonated systems. This catalytic effect was experimentally confirmed by comparing polymerization rates in reactions initiated with acid, a conventional initiator, or both. The combined initiation resulted in a higher apparent propagation rate constant (*k*_p_^app^) than the sum of the rates observed for acid‐initiated and conventional polymerizations, highlighting the synergistic effect of acid in enhancing polymerization kinetics. The higher *k*_p_^app^, coupled with reduced primary radical termination in the absence of a conventional initiator, likely explains the suppression of termination events, which improves end‐group fidelity and block copolymer synthesis. Further support for the mechanism was found in the scope of monomers amenable to acid‐triggered polymerization. While (meth)acrylic monomers were successfully polymerized under acidic conditions, with an associated increase in polymerization rate, styrene, resistant to protonation and prone to mayo‐type autoinitiation [144] showed no significant differences in polymerization when heated with or without acid. This observation aligns with the proposed protonation‐dependent pathway.

**FIGURE 8:**
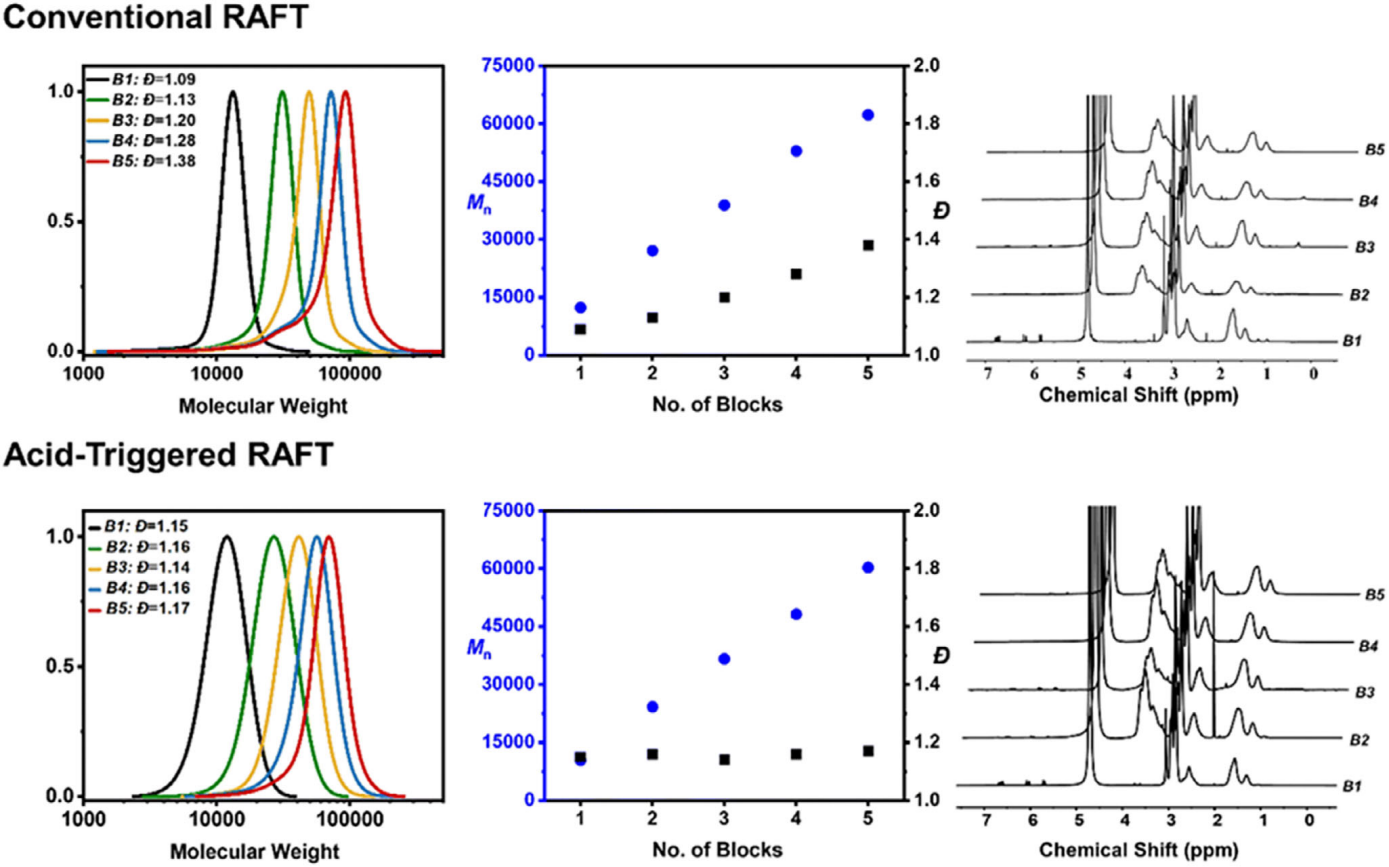
A comparison between conventional and acid‐triggered RAFT polymerization in the synthesis of pentablock copolymers. Figure adapted with permission [145]. Copyright 2024, Royal Society of Chemistry.

The high end‐group fidelity resulting from the rate acceleration and suppression of termination events observed in RAFT polymerizations in the presence of acid has recently been utilized to improve the synthesis of multiblock copolymers [145]. Addition of small amounts of acid resulted in a fourfold reduction in the overall radical initiator concentration required to achieve quantitative monomer conversion, facilitating the synthesis of a wide range of well‐defined multiblock copolymers with varying degrees of polymerization (DP) per block. The acid effectively enhances the propagation rate, allowing for the minimization of initiator concentration (Figure 8). In all cases, near‐quantitative monomer conversion (>97%) was observed for each iterative block addition step. As a result, high molecular weight multiblock copolymers were successfully synthesized, with blocks having DP values of 100, 200, and even 500, all exhibiting livingness above 90%. Furthermore, the synthesis of icosablock copolymers was achieved with a remarkably low dispersity (*Ð* = 1.16), underscoring the high level of control afforded by this method. This approach also demonstrated versatility, as it enabled the successful polymerization of multiblocks composed of methacrylates, acrylates, and acrylamides, showcasing the broad applicability of acid‐triggered RAFT polymerization across different monomer classes.

PI polymerization under visible light is an attractive, initiator‐free strategy for controlled radical polymerization, but it often suffers from prolonged induction periods, slow kinetics, and poor control when using high‐activity RAFT agents such as trithiocarbonates. Antonopoulou et al. recently showed that the addition of small amounts of citric acid can overcome these limitations by dramatically enhancing the efficiency of PI polymerization [146]. Addition of acid eliminated the induction period, increased the polymerization rate by over threefold, and enabled rapid consumption of the RAFT agent, yielding polymers with low dispersities (*Ð* ≈ 1.1) and high degrees of polymerization (up to DP = 3000) in just a few hours. DFT calculations revealed that protonation of the RAFT agent lowers the triplet bond dissociation energy and shifts the excited‐state energetics to suppress non‐productive decay pathways, thereby promoting efficient homolysis. This acid‐enhanced PI strategy proved broadly applicable to acrylamides, acrylates, and methacrylates, and enabled efficient synthesis of well‐defined block copolymers, establishing it as a powerful tool for visible‐light‐mediated RAFT polymerization.

Acid‐triggered RAFT polymerization remains in its infancy, with much still to be understood about its full potential. The rate enhancement observed under acidic conditions is not limited to just RAFT polymerization but appears to extend to free radical polymerization as well, suggesting broader applicability in controlling polymerization kinetics. Additionally, the suppression of termination events in acid‐triggered systems, though experimentally observed, has not been rigorously investigated in terms of kinetics or kinetic modelling. A deeper understanding of how acid influences termination pathways and radical lifetimes is crucial to optimizing this process. Future research will also need to address key questions such as the full scope of monomers and reaction conditions that benefit from acid‐triggered initiation/rate‐enhancement. The catalytic effect of acids could potentially offer further synergies when combined with other state‐of‐the‐art RAFT techniques.

## 3 RAFT in Unique Environments

### 3.1 Biological Settings

**FIGURE 9:**
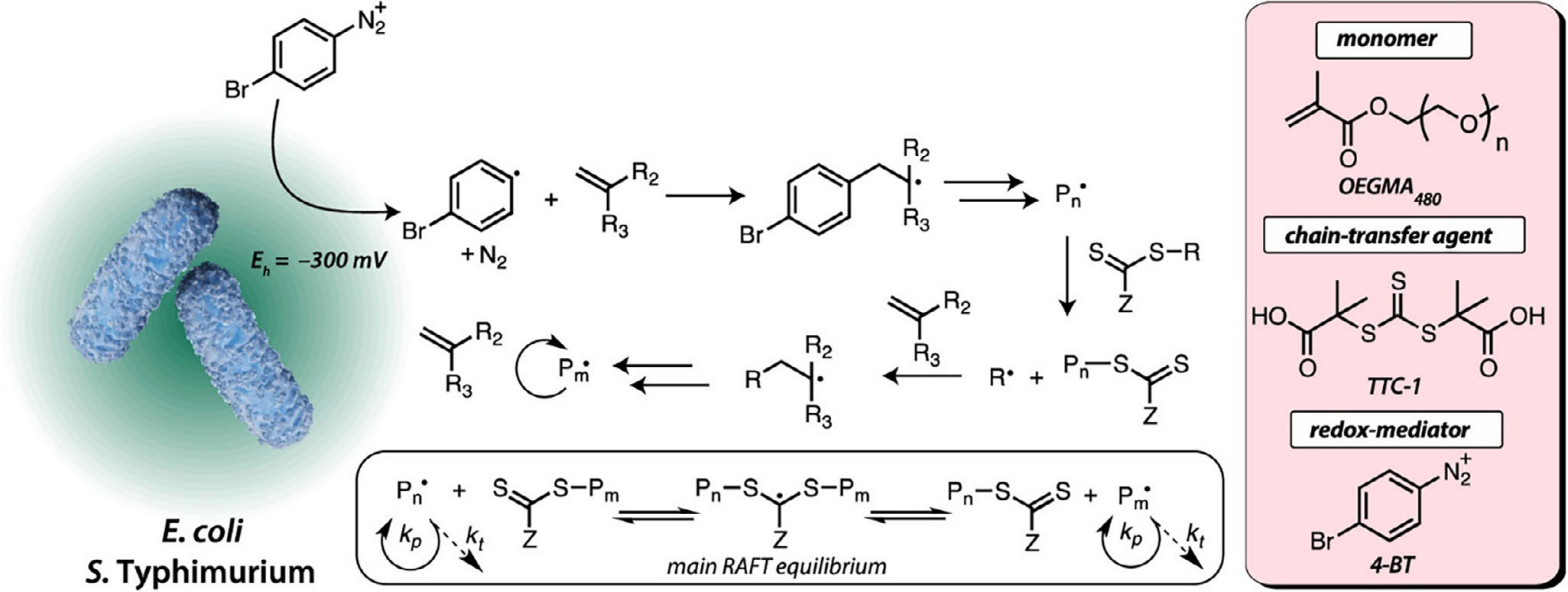
Controlled radical polymerization (BacRAFT) facilitated by the terminal electron flux of E. coli (strain MC4100) and S. Typhimurium (strain TAS2010). The reducing potential generated by growing bacteria activates the redox‐active diazonium salt (4‐bromobenzenediazonium tetrafluoroborate (4‐BT)) to furnish a carbon‐centered aryl radical, which subsequently initiates a controlled radical polymerization of methacrylate monomers (OEGMA) in the presence of a chain‐transfer agent (TTC‐1) via RAFT. Adapted with permission [147]. Copyright 2020, American Chemical Society.

The incorporation and use of living organisms to support the production of RAFT polymers is an exciting new field. In the past few years, there have been a couple of studies on investigating the use of bacteria to help initiate RAFT polymerization. This includes the Qiao group using bacteria to control the polymerization by reducing aryl diazonium salts to aryl radicals, allowing for the living polymerization of a methacrylate monomer as shown in Figure 9 [147]. While Bennett et al. [128] used an iron‐reducing bacterium and glucose oxidase to develop a Fenton‐like RAFT polymerization that possessed narrow molecular weight distributions (*Ð* = 1.12), with the bacterial cells being able to be recycled for several polymerization reactions. Geng and co‐workers also investigated the ability for polymers to be synthesized intracellularly in cancer cells by PET‐RAFT in a controlled manner and showed that this polymer prodrug approach was able to significantly inhibit tumour growth [148]. Wan et al. [149] meanwhile, used a biologically‐driven RAFT polymerization method through the addition of a NADH coenzyme to reduce RAFT agents for the purpose of preparing an electrochemical platform for the detection of antibody drugs.

### 3.2 Oxygen‐Tolerant RAFT

In recent years, oxygen‐tolerant reversible deactivation radical polymerization (RDRP) has gained significant attention for simplifying polymerization processes [150]. Among the various oxygen‐tolerant systems developed for RAFT polymerization, PET‐RAFT has demonstrated remarkable oxygen tolerance [27]. Early studies utilizing catalysts such as Ir(ppy) and Ru(bpy) revealed that polymerization could proceed without deoxygenation by consuming oxygen through its conversion to superoxide. However, these systems were hindered by prolonged inhibition periods, complicating the achievement of predictable polymerization kinetics. The introduction of zinc tetraphenylporphyrin (ZnTPP) as a photocatalyst effectively addressed this limitation [32]. ZnTPP enhanced oxygen conversion into singlet oxygen through triplet‐triplet annihilation, significantly shortening inhibition periods in dimethyl sulfoxide (DMSO). The singlet oxygen was quenched by DMSO, forming dimethyl sulfone, or reacted with other quenchers such as thioethers or ascorbic acid. Importantly, the polymerization kinetics were only slightly affected by the presence of oxygen, and the resulting polymers exhibited similar molecular weights and dispersities to those prepared under deoxygenated conditions. This advancement greatly improved the efficiency and predictability of oxygen‐tolerant PET‐RAFT polymerization in organic solvents. However, until recently, PET‐RAFT oxygen‐tolerant polymerization was largely confined to organic solvents, particularly DMSO. Efficient aqueous systems were later developed using zinc tetrakis(4‐sulfonatophenyl)porphyrin (ZnTTPS) or Eosin Y (EY) as photocatalysts [151]. These systems achieved good control over polymerization by incorporating additives such as triethanolamine (TEOA) or ascorbic acid to rapidly quench oxygen and form reactive oxygen species. The combination of EY and TEOA results in the formation of superoxide, while EY and Ascorbic Acid resulted in the formation of hydrogen peroxide and hydroxyl radicals [151]. While the formation of these reactive oxygen species does not alter the chemical structures of the resulting polymers, it may, in some cases, have unexpected effects on monomers or biomacromolecules. This is particularly relevant when PET‐RAFT is employed for the preparation of protein‐polymer conjugates [151].

Another strategy to confer oxygen tolerance involves the use of enzymes capable of converting oxygen into hydrogen peroxide. This approach was first introduced by Yagci's group, who utilized glucose oxidase (GOx) to consume oxygen [152]. Subsequently, this method was adopted by Stevens’ group and An's group [153] for RAFT polymerization. Chapman and co‐workers were the first to demonstrate the use of enzymes to facilitate RAFT polymerization under open‐vessel conditions [154]. In their approach, GOx acted as a deoxygenation agent, while a thermal initiator was employed to generate radicals necessary for the RAFT process. Building on this concept, An and co‐workers developed an alternative approach that replaced the thermal initiator with a second enzymatic reaction to produce radicals [155]. Specifically, they employed horseradish peroxidase (HRP) to catalyze the oxidation of acetylacetone in the presence of hydrogen peroxide (H_2_O_2_), resulting in the formation of radicals suitable for initiating RAFT polymerization. The main advantage of these enzymatic reactions is that they can efficiently be performed in water at relatively low concentrations of enzymes and at room temperature.

In addition to PET‐RAFT and enzyme‐RAFT polymerization, other oxygen‐tolerant RAFT polymerization mechanisms have been explored. Photoiniferter (PI) RAFT polymerization, as well as thermal or photoinitiated RAFT polymerization, achieves partial oxygen tolerance through the consumption of oxygen by radicals—a process often referred to as “polymerization through oxygen.” In this approach, generated radicals react with oxygen to form less reactive peroxide radicals. While this method is effective, it typically requires high concentrations of initiators or RAFT agents to fully consume oxygen, leading to a loss of end‐group fidelity and a slight increase in dispersity. This limitation was overcome by the addition of tertiary amines to PI RAFT, which further improves oxygen tolerance by converting oxygen into superoxide through a proposed mechanism [156]. For instance, Qiao and coworkers demonstrated that incorporating tertiary amines into the PI RAFT process enabled polymerization to proceed without the need for deoxygenation. Gibson and coworkers harnessed this enhanced oxygen tolerance to develop a high‐throughput process for creating a diverse library of polymers [157].

**FIGURE 10:**
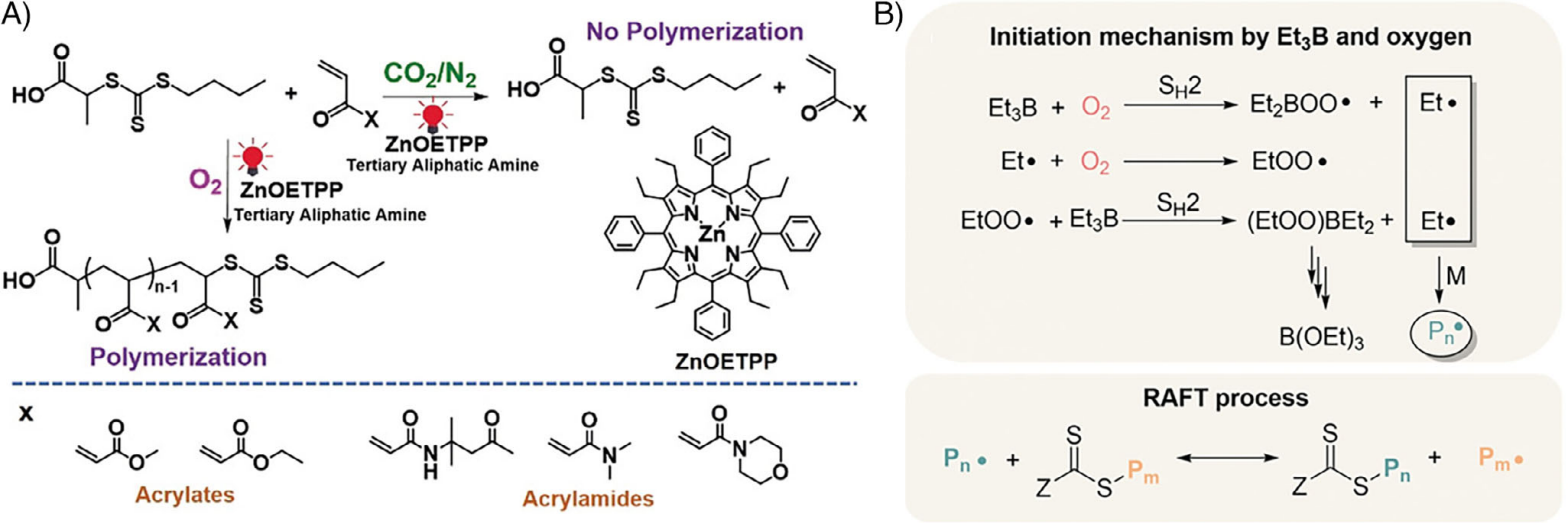
Representative scheme of the two distinct strategies for oxygen facilitated RAFT polymerization; A) Mechanism of ZnOETPP‐catalyzed PET‐RAFT photopolymerization that proceeds only in the presence of a tertiary aliphatic amine and oxygen. Adapted with permission [55]. Copyright 2019, John Wiley and Sons; B) Mechanism for radical initiation by triethylborane and oxygen. Adapted with permission [158]. Copyright 2018, John Wiley and Sons.

A novel paradigm has recently emerged in which oxygen is actively utilized as a reactant, playing a pivotal role in activating RAFT polymerization. In this approach, polymerization is entirely oxygen‐dependent and cannot proceed in its absence. Two distinct strategies have been developed under this paradigm: i) The first strategy employs a photocatalytic approach to convert oxygen into active radical species (Figure 10A). Researchers have demonstrated that specific photocatalysts, such as zinc(II) (2,3,7,8,12,13,17,18‐octaethyl‐5,10,15,20‐tetraphenylporphyrin) [55] and specific COFs [45], in combination with triethylamine (TEA), enable RAFT polymerization by leveraging oxygen as a critical reactant. Remarkably, polymerization does not occur in oxygen‐free environments, but the introduction of oxygen facilitates successful polymerization. This counterintuitive mechanism relies on the conversion of oxygen into active intermediates (hydroxyl radicals), highlighting its indispensable role in this process. ii) The second strategy, initially reported by Pan's group, capitalizes on the unique reactivity of alkylboranes (Figure 8B) [158, 159]. Alkylboranes (BR_3_), which are pyrophoric, generate radicals upon reacting with oxygen, making them highly effective initiators for polymerization. For example, Pan and colleagues utilized triethylborane (Et_3_B) and oxygen to generate ethyl radicals, which successfully initiated RAFT polymerization. Notably, this polymerization system could be switched “on” and “off” by alternately introducing and removing air (or oxygen) from the reaction mixture. Building on this concept, Magenau and coworkers proposed an alternative method that uses air‐stable alkylborane/amine complexes [160]. In this approach, the addition of a deblocking agent, such as carboxylic acid compounds, activates the polymerization by enabling the reaction of borane with oxygen. This modification improves the stability and practical applicability of the system while retaining its oxygen‐dependent functionality.

### 3.3 RAFT in Deep Eutectic Solvents (DES) and Ionic Liquids (IL)

Alternative solvents for RAFT polymerization have been explored to offer better reaction conditions and kinetics, as well as expand the range of applications for these systems. In this context, deep eutectic solvents (DES) and ionic liquids (IL) have been explored as non‐volatile alternatives for organic solvents in RAFT polymerization.

**FIGURE 11:**
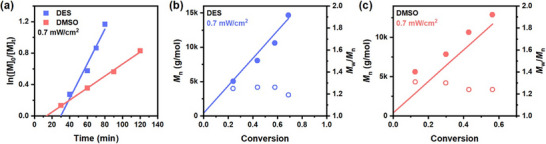
Open‐to‐air photoiniferter polymerization using either the DES or DMSO under blue light (λ_max_  = 465 nm). (a) ln([M]_0_/[M]_t_) versus time under 0.7 mW/cm^2^ of blue light. (b) and (c) M_n_ (filled circle) and M_w_/M_n_ (open circle) versus conversion for the DES and DMSO under 0.7 mW/cm^2^ of blue light. The concentration of MMA was 30 wt %, and the molar ratio of [MMA]/[CTA1] was 200:1 for all experiments. Reproduced with permission [25]. Copyright 2021, American Chemical Society.

In 2021, Yu and coworkers reported the use of deep eutectic solvents in photo‐RAFT polymerization (Figure 11) [25]. The DES composed of tetrabutylammonium chloride, and ethylene glycol. This non‐volatile reaction medium provided facile photo‐RAFT conditions for (meth)acrylates, acrylamides, and styrene. Oxygen tolerance was achieved in PI‐RAFT via a polymerising through mechanism, and in PET‐RAFT through the use of tertiary amines as co‐catalyst. The improved reaction kinetics and control on MW and chain‐end fidelity was attributed to the higher photostability of RAFT agent in DES, and an increased *k*_p_^app^.

Ionic liquids as alternative solvents have also been explored over the last two decades, and their effect on polymerization and possible mechanisms of action have been explored [161, 162, 163, 164]. Prior studies on free radical polymerization systems discuss the rate enhancement effect of ionic liquids on polymerization [165]. The main reason for rate enhancement and control provided by ionic liquids has in the past been attributed to increased polarity, increased stability of reaction intermediates, and the protected radical effect which decreased termination and helped with the formation of monomer domains. In 2023 [23], Qiao and coworkers reported the effect of ionic liquids on visible blue‐light mediated PI‐RAFT of aqueous acrylamide and acrylate systems. A rate increase in polymerization was observed when 1‐ethyl‐3‐methylimidazolium ethyl sulfate [EMIM][EtSO_4_] ionic liquid was used as (co‐)solvent with water. It was reported that at least 50% IL was required to observe the rate enhancement effect under deoxygenated conditions.

**FIGURE 12:**
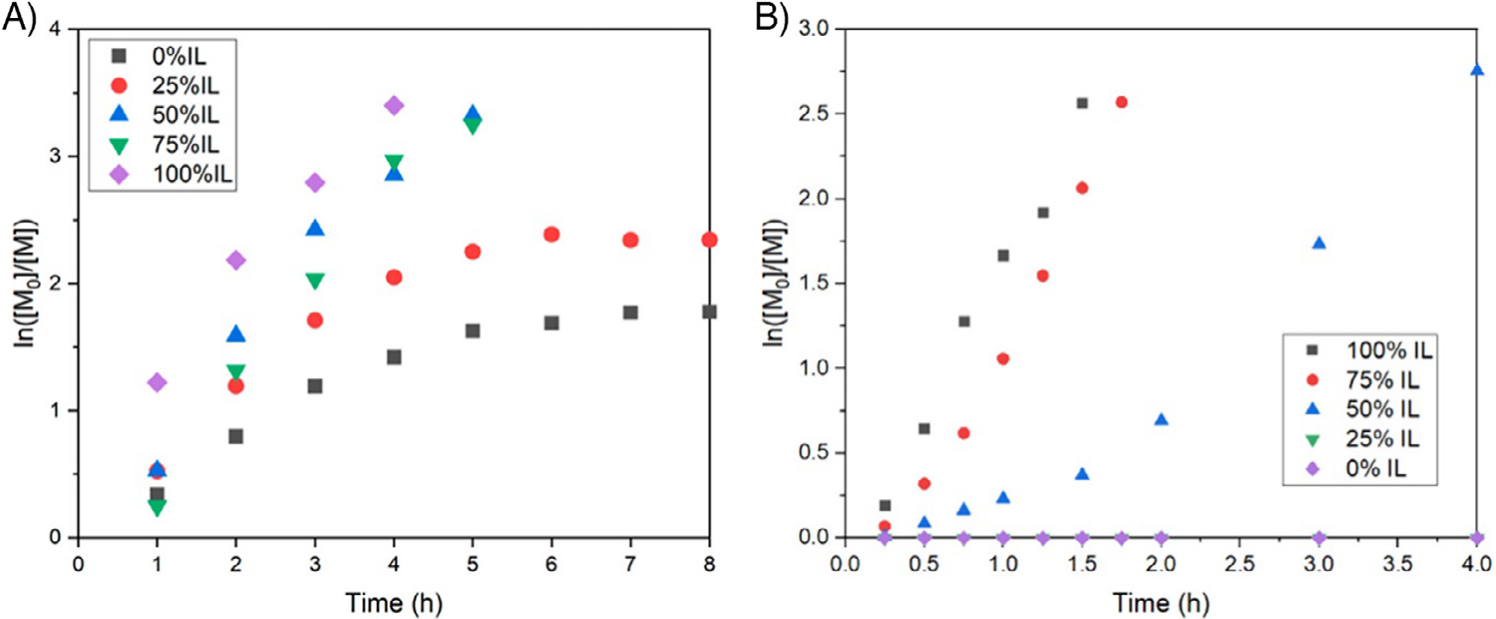
A) Polymerization kinetics of MMA in the [BMIM][PF_6_]:DMSO solvent system with varying volume fractions of IL under visible blue light at 2 M monomer concentration and [M_0_]/[CDTPA] = 200. Reproduced with permission [24]. Copyright 2024, American Chemical Society. B) Polymerization kinetics of DMA in different IL/H_2_O ratios under blue light irradiation with DMA/CTMPA/EY 200:1:0.1 under nondegassed conditions. Reproduced with permission [166]. Copyright 2024, American Chemical Society.

Later, the same group [24] expanded on this work to organic systems, demonstrating rate enhancement for the polymerization of methacrylates in IL/DMSO mixtures (Figure 12A). They also demonstrated oxygen tolerance in the system, while showcasing the recyclability of IL. They then reported the use of IL/DMSO and IL/water solvent systems as reaction media for the PET‐RAFT polymerization of acrylamides using the organic catalyst EY (Figure 12B) [166]. Under green and blue visible light irradiation at low irradiance (less than 3 mW/cm^2^), they were able to successfully synthesize highly living and controlled polymers in the presence of oxygen without the need for tertiary amines as cocatalysts with enhanced reaction rates. The benefit of IL as reaction (co‐)solvent was further demonstrated by successfully polymerising acrylamides using ultra‐low concentrations of EY (0.001 equiv. with respect to RAFT agent) without the need for co‐catalysts in the presence of oxygen. With the incorporation of IL at levels as low as 25%, efficient polymerization was achieved, which was attributed to the higher stability of RAFT agent in the presence of IL, enhanced apparent propagation rates, and possible “trapping” mechanisms resulting in a faster polymerization and oxygen tolerance. In 2024 [167], natural deep eutectic solvents that consist of organic acids, amino acids, and sugars were used both as reaction solvent, and in other cases mixed as solids with the monomer NIPAM to subsequently polymerize to yield natural deep eutectic solvent‐based polymer. Thermal and UV‐based photo‐initiated free radical and RAFT polymerization were performed, with an enhanced reaction kinetic in the presence of oxygen. Collectively, ILs, DESs, and related green solvents offer advantages of improved kinetics, recyclability, and non‐volatility, making them promising media for advanced polymerization and 3D printing applications. Building on these advances, polymerizable eutectics have been combined with bis‐RAFT agents to produce visible‐light 3D printable resins with high strength, improved adhesion, thermoresponsive behaviour, and excellent shape fidelity, highlighting their potential in biomaterials and actuator design [168].

## 4 Advanced and Emerging Applications of RAFT

### 4.1 Alternative RAFT‐Like Chain Transfer Agents

#### 4.1.1 Sulfur‐Free

**FIGURE 13:**
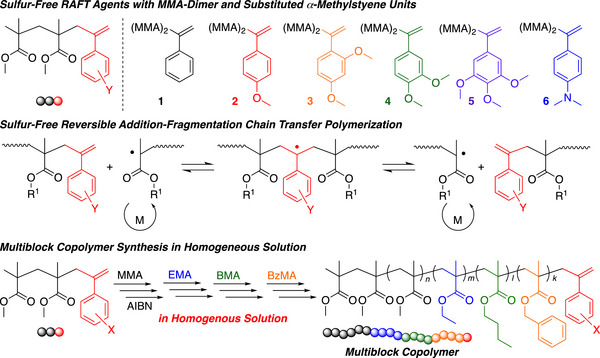
Sulfur‐free radical RAFT polymerization of methacrylates using exo‐olefin‐based RAFT agents. Adapted with permission [181]. Copyright 2022, John Wiley and Sons.

One of the major challenges in radical RAFT polymerization is the development of efficient sulfur‐free RAFT agents that can undergo rapid, reversible addition‐fragmentation chain transfer processes to produce colorless and odorless polymers with controlled molecular weights. RAFT polymerization is typically performed using TCT compounds, but the usefulness of a reversible addition‐fragmentation mechanism for controlled radical polymerization was reported earlier with poly(methyl methacrylate) (PMMA) macromonomer with *exo*‐olefin terminal prepared by the cobalt‐mediated catalytic chain‐transfer polymerization of MMA [169, 170]. Although the chain‐transfer constant (*C*_tr_) of the *exo*‐olefin terminal was relatively low in comparison with those of TCT compounds [171], the PMMA macromonomer sufficiently works to give methacrylate block copolymers with controlled molecular weights under emulsion conditions, where the supply of monomers from the droplets in the aqueous phase apparently slows propagation in comparison with chain transfer. Indeed, the sulfur‐free PMMA macroRAFT agents were applied for the synthesis of methacrylate multiblock copolymers under emulsion conditions by Haddleton, Davis, Anastasaki, and coworkers in 2017 [172]. Such heterogeneous emulsion polymerizations were further used to prepare various block copolymers and latexes [173, 174, 175, 176, 177, 178, 179, 180]. In 2022, Satoh et al. reported the synthesis of *exo*‐olefin‐based RAFT agents (R–CH_2_C(= CH_2_)Z) possessing an MMA dimer structure as the R group and a phenyl group with electron‐donating substituents such as *p*‐methoxy group as the Z group for the sulfur‐free RAFT polymerization of methacrylates in homogeneous solutions (Figure 13) [181]. Because the structure of the –CH_2_C(= CH_2_)PhOMe unit is similar to that of *p*‐methoxystyrene, which has a high reactivity to electron‐deficient polymethacrylate radicals, the *C*_tr_ values increased, enabling the synthesis of colorless and odorless multiblock polymethacrylates upon the continuous addition of various methacrylates in organic solvents.

#### 4.1.2 Selenium

**FIGURE 14:**
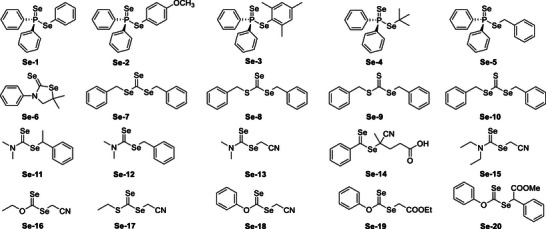
Selenium‐based RAFT agents.

As analogous of sulfur‐based RAFT agents, selenium‐based RAFT agents also demonstrate excellent controllability over the polymerization of various vinyl monomers. Moreover, the synthesized selenium‐containing polymers have significant potential for biomedical applications due to their redox sensitivity [182]. Diselenide‐containing polymers can be easily prepared through the aminolysis of diselenocarbonate‐terminated polymers and a spontaneous oxidation coupling reaction in air [183]. To date, various selenium‐based RAFT agents have been designed and synthesized (Figure 14) [184, 185, 186, 187, 188, 189, 190]. Lee et al. reported the first use of phosphinodiselenoic acid esters (**Se‐1**∼**Se‐4**) as RAFT agents for the polymerization of styrene, although the resulting polymers exhibited a relatively broad polydispersity (ranging from 1.8 to 4.8). Later, a similar phosphinodiselenoic acid ester (**Se‐5**) was synthesized for styrene polymerization under UV–vis irradiation, yielding polymers with a polydispersity ranging from 1.5 to 2.0. Various selenium‐based RAFT agents (**Se‐6**∼**Se‐20**) have been designed and synthesized, including (cyclic) diselenocarbamates, selenodithiocarbonate, diselenothiocarbonate, triselenocarbonate, and (xanthate‐type) diselenocarbonates. Notably, **Se‐20** showed excellent control over a wide range of vinyl monomers, including styrene, acrylates, vinyl esters, and methyl methacrylate, making it a ‘universal’ RAFT agent [188]. In most cases, the selenium‐based RAFT agents demonstrated similar controllability to their sulfur‐based counterparts. In a recent theoretical study [191], it was found that dithiocarbamates and diselenocarbamates with the same R and Z groups exhibit similar reactivities RAFT agents. However, the radical adducts formed from diselenocarbamates are more prone to intermediate radical termination than those formed from dithiocarbamates, resulting in significant retardation of the polymerization process.

### 4.2 Ultrahigh Molecular Weight

Ultra‐high‐molecular‐weight (UHMW) polymers (molecular weight ≥10⁶) exhibit exceptional material properties [193, 194] but are challenging to synthesize due to the competition between chain propagation (*kp*) and radical termination processes [14]. The advent of photo‐mediated RAFT techniques, particularly UV‐mediated photo‐iniferter RAFT polymerization, has significantly advanced the synthesis of UHMW polymers [195].

Sumerlin et al. demonstrated the potential of photolysis of trithiocarbonate (TTC) compounds under aqueous conditions to achieve UHMW acrylamides [196], leveraging reversible termination pathways for enhanced control. This methodology was later extended to a range of monomers in organic solvents [16]. Recent studies have widely adopted the “gel polymerization” concept [197], where increased solution viscosity reduces termination rates by limiting radical encounters. Others have employed solvents to accelerate *kp* and further improve polymerization efficiency [198].

To address scaling challenges associated with high viscosities in UHMW polymer synthesis, Sumerlin introduced mini‐emulsion photo‐iniferter RAFT polymerization, which confines reactions within dispersed particles [15]. This innovation reduces overall viscosity and facilitates continuous‐flow processes [199]. More recently, this strategy was applied to synthesize UHMW ABA triblock copolymers featuring stimuli‐responsive terminal blocks [200].

Chen et al. expanded the field by employing PET‐RAFT polymerization to prepare UHMW fluorinated copolymers, including alternating copolymers of fluorinated maleimides with styrene, vinyl ether, and *N*‐vinylpyrrolidone [201]. Notably, UHMW fluorinated methacrylate polymers were synthesized by exploiting the limited fragmentation efficiency of macro‐CTAs, achieving higher degrees of polymerization than predicted by the monomer/CTA ratio [150].

**FIGURE 15:**
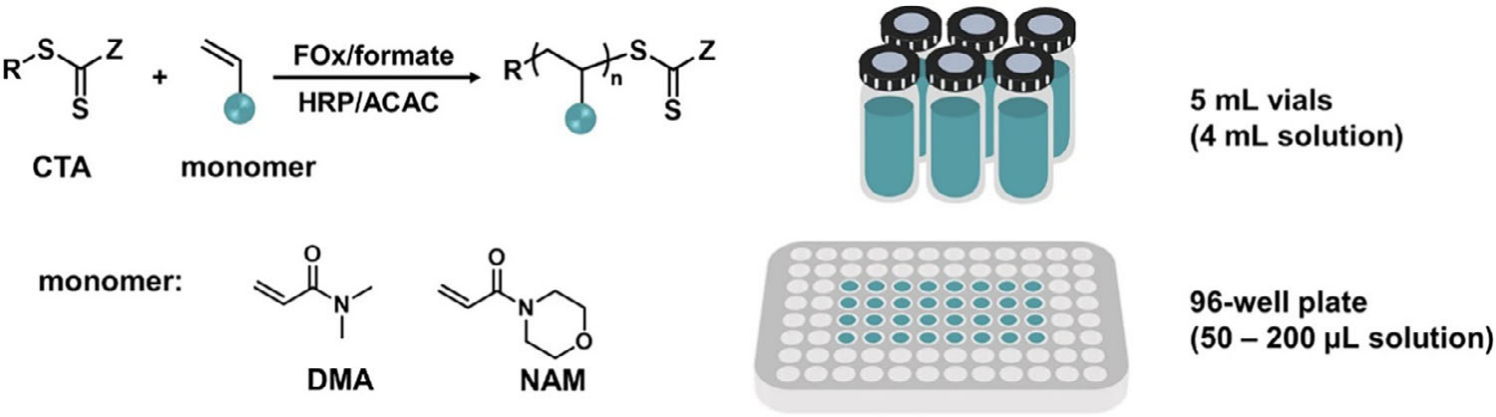
Schematic representation of synthesis of UHMW polymers in ultralow volumes via FOx‐HRP enzymatic cascade catalyzed RAFT polymerization and chemical structures of monomers. Adapted with permission [192]. Copyright 2022, John Wiley and Sons.

Enzyme‐mediated RAFT polymerization has also progressed in UHMW synthesis [202]. An and co‐workers recently prepared UHMW polymers in ultralow volumes (∼50 µL) using 96‐well plates under open‐air conditions, highlighting its potential for high‐throughput screening (Figure 15) [192]. Pan et al. made advancements in organoborane‐initiated RAFT polymerization, demonstrating its utility for UHMW synthesis even under initial air exposure [203].

Despite these advancements, challenges persist in achieving well‐defined UHMW homopolymers from unconjugated monomers with RAFT polymerization techniques. The highest reported molecular weight for UHMW vinyl ester under optimized PET‐RAFT conditions is *M*_n_ ∼350 kDa, though control remains poor (*Ð* ∼1.9) [204].

More recently, efforts have shifted toward tuning the molecular dispersity of UHMW polymers. In a recent study, An employed a tetrafunctional, switchable RAFT agent to prepare UHMW star polymers and investigate the impact of dispersity on rheological behaviour [205]. In another report, dispersity was modulated by mixing CTAs in a photoenzymatic RAFT polymerization. The resulting hydrogel showed that the storage modulus (G′) scaled with molecular weight but peaked at intermediate *Ð* values [206].

### 4.3 Continuous Flow

Since our previous update, there has been a surge in the literature on both the monitoring of the polymer synthesis in continuous flow and leveraging the advantages of various non‐traditional RAFT activation methods within flow.

The combination of flow chemistry and inline/online analysis to create a streamlined model for polymer synthesis has been of great interest. The investigation and following of polymer properties within the flow set‐up has been achieved by the incorporation of characterization instruments including inline NMR [19, 207, 208], inline diffusion‐ordered NMR [209], a combination of inline NMR and online GPC [210, 211], in situ SAXS [212], and even the ability for inline formation and purification of self‐assembled block copolymers has been investigated [213, 214].

**FIGURE 16:**
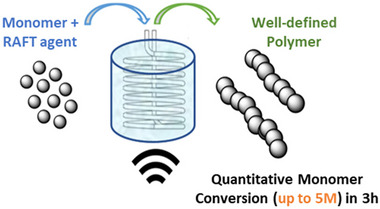
Sono‐RAFT in continuous polymerization that employs stainless‐steel tubing to improve surface area and enhance cavitational intensity, resulting in quantitative monomer conversions of up to 5 M. Adapted with permission [64]. Copyright 2023, American Chemical Society.

This coupled with the incorporation of various RAFT polymerization methods such as ultrasound [64] (Figure 16), PET‐RAFT [215], surface initiated PET‐RAFT [216], visible light induced flow cationic RAFT polymerization [107], PISA [208, 212], enzyme‐assisted photo‐PISA [217], and photoiniferter [218, 219] into the continuous flow system allows for many different chemistries, different formation of structures, and applications to be designed for with limited researcher interaction.

The continued attention in combining flow chemistry with online/inline monitoring and automated software/machine learning has been fuelled by the desire to design polymer set‐ups for high‐throughput screenings and designing of a closed‐loop system. The combination of various synthesis methods and characterization instruments would allow for consistent and robust collection of high‐throughput data, while eliminating systematic errors that might be present in single reaction setups, and increase both precision and accuracy of the reactions independent of the researcher [210, 220].

### 4.4 Automation and ML

To navigate the complex chemical space of polymeric systems for an efficient design of polymers with different targeted properties, a deep understanding of the underlying structure‐property relationships of these materials is required. Automation and high throughput experimentation can aid in rapid synthesis and testing of polymers, while the production of large databases of structures, system parameters, and properties can enable the training of machine learning (ML) algorithms for the data‐driven prediction and design of polymers for various applications. RAFT polymerization offers a valuable opportunity in this context, offering the ability to synthesize precise polymer architectures under relatively facile reaction conditions.

**FIGURE 17:**
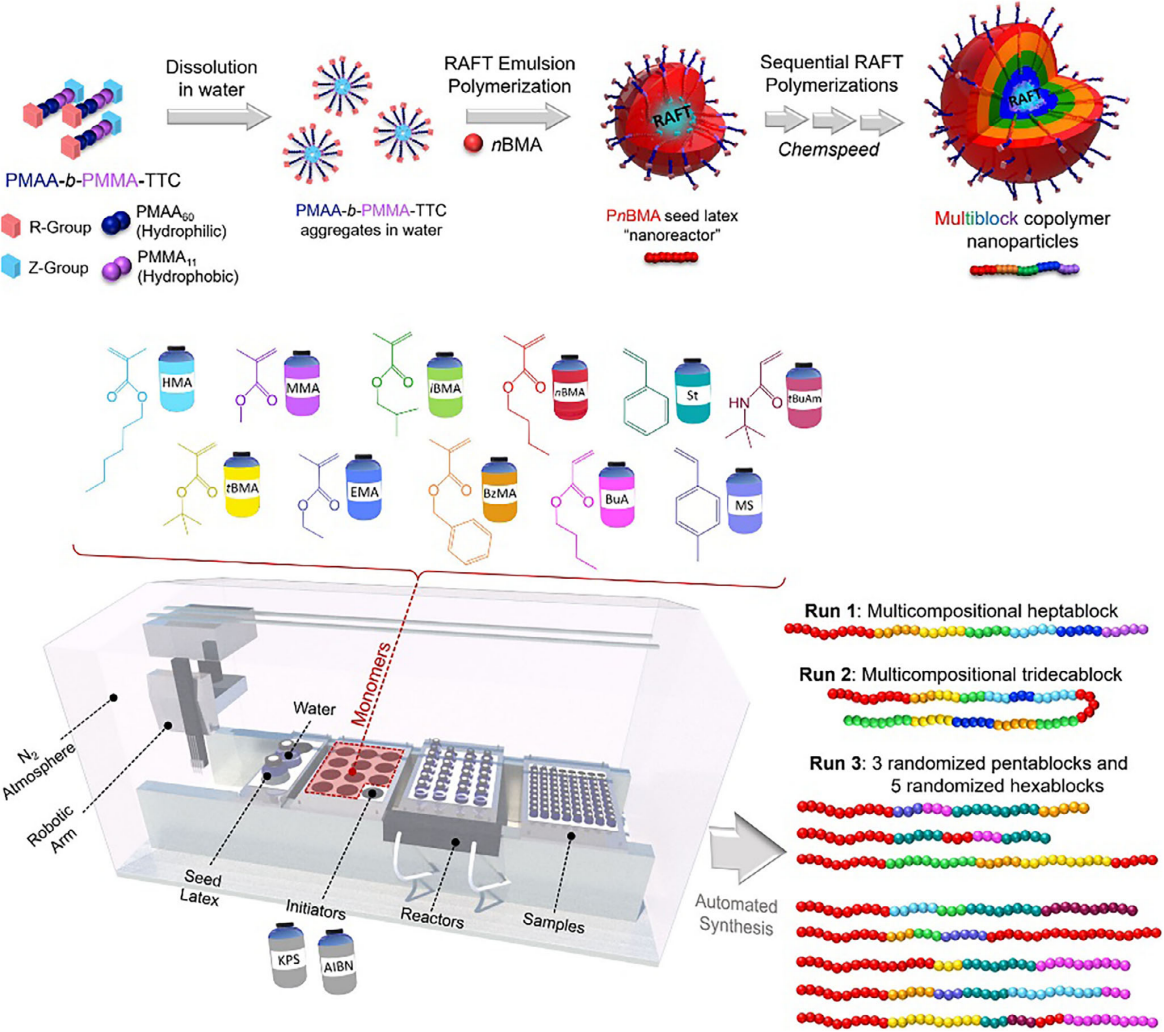
Schematic depiction of the general methodology for synthesis of the PnBMA seed latex and subsequent chain extension for the generation of multiblock copolymers, and an illustration of the Chemspeed robot employed. Reproduced with permission [224]. Copyright 2024, John Wiley and Sons.

In recent years, RAFT polymerization has been used to develop and expand automated polymer synthesis platforms for the production of polymer libraries, focusing on the precision of polymer structure and architecture in an effort to understand structure‐property relationships more accurately. Different liquid handling robots have been explored for facile synthesis and preparation of complex polymeric libraries, such as the Hamilton MLSTARlet liquid handling robot and the Opentrons OT‐2 liquid handling robot for the automated and oxygen‐tolerant synthesis of polymers using PET‐RAFT, thermal enzyme‐RAFT, and semi‐bio‐Fenton RAFT [123, 221, 222, 223]. More complex and high‐end robotic platforms such as the Chemspeed robot have also been explored to expand the range of complex polymer libraries prepared in an automated fashion, an example is shown in Figure 17 [224, 225].

To establish reliable structure‐property relationships for polymer systems, analysis data must be collected in a high‐throughput manner to enable rapid characterization of polymer libraries. Online and inline characterization tools can enable the collection of real‐time data without the need for human interaction with the robotic platform, removing the “characterization bottleneck” that has hindered the collection of large property databases for polymers. Online characterization in batch mode requires the use of complex robotic platforms equipped with robotic arms capable of preparing and transferring samples to automated test stations [226, 227]. Flow reactors offer an advantage in this context, as the sample can directly flow into instruments for analysis [210]. Inline NMR analysis in flow systems has been extensively explored in the last few years, providing valuable information on reaction kinetics [19, 207] The use of in situ and online/inline diffusion‐ordered spectroscopy (DOSY) NMR has also recently been reported for the rapid evaluation of molecular weight for RAFT polymerization systems [228, 229, 230], which offers a great advantage in comparison to the conventional time‐consuming gel permeation chromatography techniques for MW analysis.

**FIGURE 18:**
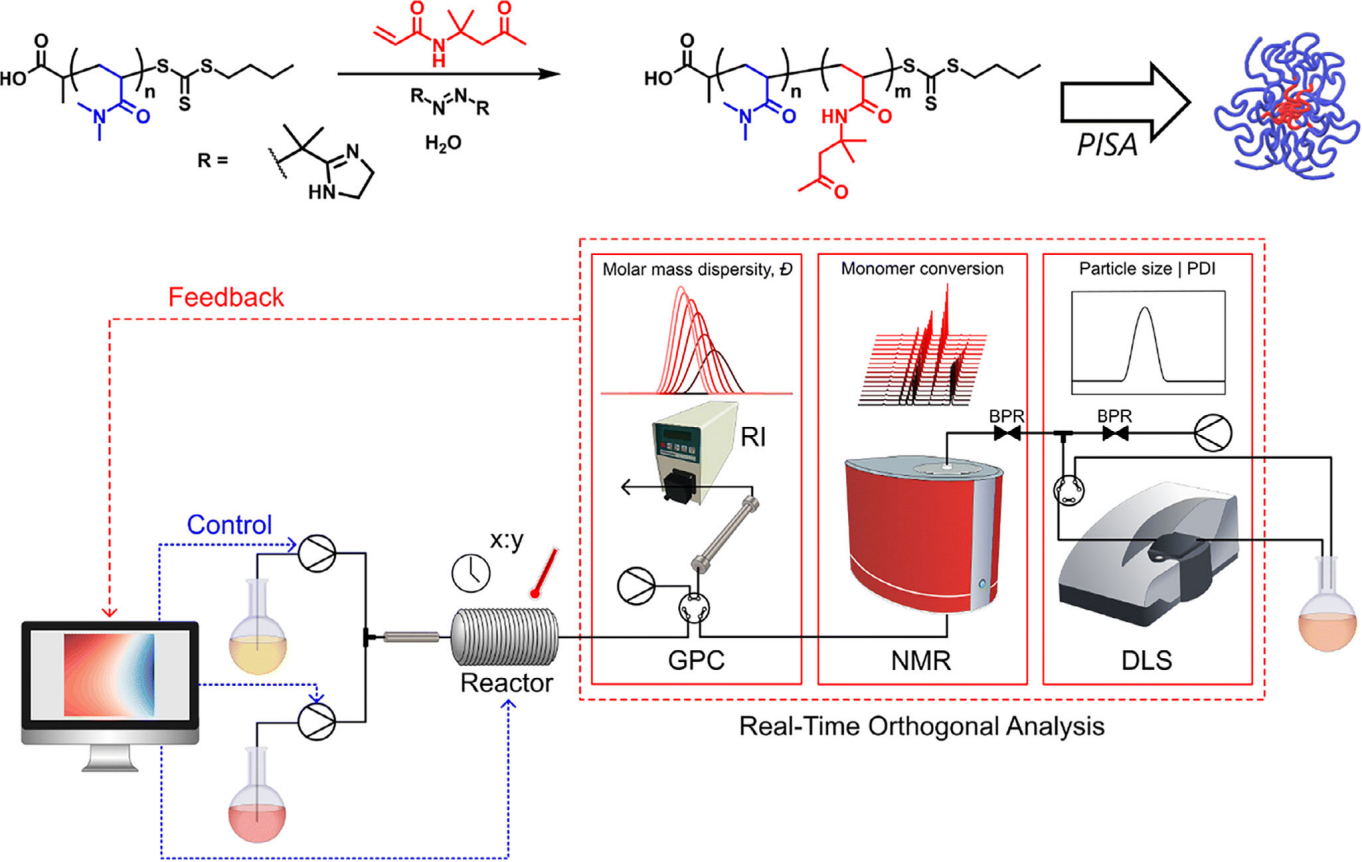
Overview of the chemistry and autonomous platform integrating a flow reactor and online gel permeation chromatography (GPC), ^1^H nuclear magnetic resonance (NMR) spectroscopy, and dynamic light scattering (DLS). Reproduced with permission [232]. Copyright 2025, The Royal Society of Chemistry.

With analysis data collected in an automated fashion, large databases can be curated, which can then be used as training datasets for ML algorithms. These algorithms can identify underlying structure‐property relationships for the prediction of polymer formulations for various applications and can drive a closed‐loop reactor system to prepare polymers with set targets. An earlier example of such a system was reported in 2019 by Junkers and coworkers [231], where a self‐optimising flow reactor was able to rapidly evaluate MW through GPC and optimize reactor conditions to reach a target MW. More recently, Warren and coworkers [232] have reported the development of a self‐driving flow reactor in which particle‐forming RAFT polymerization is explored in a fully automated fashion with data acquisition through online NMR, GPC, and DLS units (Figure 18). Use of ML algorithms enabled targeting of specific particle sizes while optimizing conversion and dispersity.

In the past five years, we have seen an increase in research focused on the training of ML models for different applications. These include using an automated flow reactor for the RAFT synthesis of block copolymer ^19^F magnetic resonance imaging (MRI) agents, utilising ML models for the design and understanding of structure‐property relationships [220], models for the prediction of antibacterial activity of synthetic polymers [233], and hybrid systems for protein stabilization and delivery [234, 235]. There have also been models designed for the multivariate optimization of chemical composition, molecular weight of polymers, moving away from the one‐variable‐at‐a‐time approach to more complex multi‐objective optimization of polymer systems [236, 237].

Perhaps the most important limitation for the ML‐driven design of polymer systems is the lack of comprehensive datasets. Data from published literature is often limited and biased toward successful experiments, while ML models require access to both successful and unsuccessful experimental conditions as training data to design reaction conditions efficiently [238]. Computational simulation and modelling can generate in‐silico datasets in conjunction with automated synthesis platforms to reinforce the datasets required for training of ML models [239]. Generation of FAIR databases (data that offers findability, accessibility, interoperability, and reusability) must be the focus of future work in this field to ensure access to robust ML models for the prediction of polymer structure‐property relationships.

### 4.5 Dispersity Control

Dispersity (*Ð*) is a key parameter in polymer science, representing the width of the molecular weight distribution (MWD) of a polymer sample. Dispersity plays a critical role in determining the properties of polymeric materials, e.g., influencing various physical properties, self‐assembly behaviour, and the characteristics of polymer brushes. Due to these broad implications, dispersity can be considered a valuable parameter that could be fine‐tuned to achieve specific material properties. In RAFT polymerization, dispersity typically serves as an indicator of the polymerization's success. A low dispersity value (Ð < 1.30) can be indicative of a well‐controlled process with high end‐group fidelity. In contrast, high dispersity values might suggest excessive chain termination or other side reactions, reducing the polymer's utility for further functionalization. However, this is only a general rule of thumb, and in recent years various techniques have been developed to give high dispersity RAFT polymers which contain a high proportion of active end‐groups.

The simplest method of tuning the MWD is to mix pre‐synthesized polymers in different ratios [240]. However, this process can be time‐consuming, requiring the synthesis of multiple batches of polymers, and often results in multimodal MWDs. In 2017, Boyer and coworkers developed a PET‐RAFT system in combination with flow processes to control MWDs by continuous in situ mixing [241]. Through altering the flow rates of the different reactant streams, the concentrations of individual components (CTA, monomer, etc.) could be tuned to produce polymers with targeted molecular weights. In situ mixing of these fractions in different ratios allows for customizable MWDs to be obtained. The groups subsequently expanded the combination of PET‐RAFT and flow achieve the synthesis low dispersity block copolymer mixtures with tailored composition gradients [242, 243]. These approaches, although streamlined compared to batch mixing, cannot directly target a specific MWD shape, and thus experimental optimization is still necessary. In 2019, Junkers overcame this by developing a systematic framework for predicting the shape of MWDs a priori to experimental mixing, allowing for a designed MWD to be targeted directly [244]. To overcome this, Boyer and co‐workers introduced a mathematical approach enabling the preparation of tunable molecular weight distribution and dispersity [245].

Direct synthesis of polymers with a range of dispersities by RAFT polymerization could theoretically be achieved by utilizing individual CTAs with differing activities [246]. However, this would require the synthesis of multiple CTAs with precise differences in activity. In 2020, Anastasaki and co‐workers instead proposed that *Ð* could be tuned by mixing of just two CTAs with differing activity in different ratios [247]. In a model system with MMA, polymerization with highly active dithiobenzoate yielded a polymer with *Đ* ∼ 1.13, whereas a less active dithiocarbamate yielded *Đ* ∼ 1.65. Conducting polymerizations with mixtures of these two CTAs in different ratios allowed for the dispersity to be tuned between these two extremes, with monomodal MWDs in all cases. Selecting appropriate CTA pairs also allowed for similar dispersity control for acrylates, styrene, acrylamides, vinyl ketones, and vinyl acetate. The concept of mixed RAFT agents to tune dispersity has also been applied to PET‐RAFT polymerization, with similar degrees of precision [248].

**FIGURE 19:**
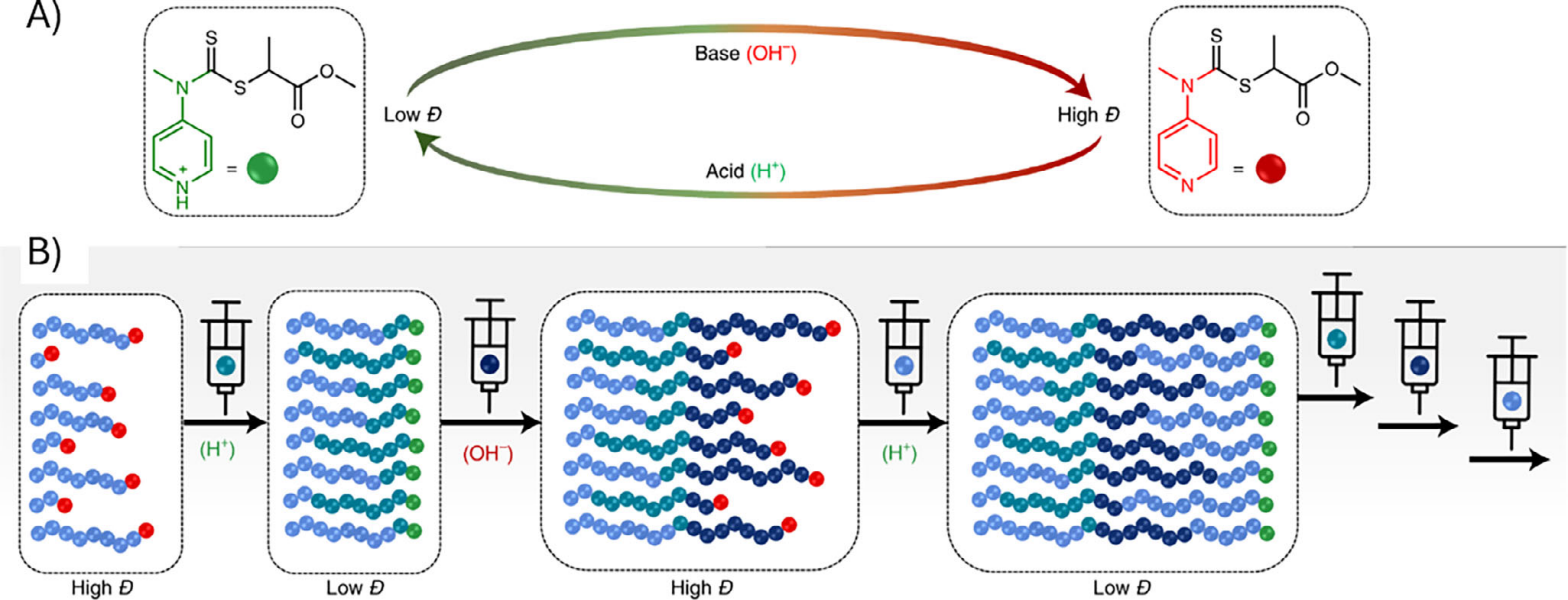
Representative scheme for A) Control over RAFT agent activity with addition of acid or base switching RAFT agent from low to high activity as de/protonation of the aromatic amine increases/ decreases the electron‐withdrawing ability; and B) Multiblock copolymers with alternating high and low dispersities through addition of acid and base. Green circles, protonated RAFT agents; red circles, deprotonated RAFT agents. Adapted with permission [249]. Copyright 2021, Springer Nature.

Although polymer blending and the mixed RAFT agent approach are versatile, they offer limited control over the dispersity of individual blocks in block copolymers, where only tuning of the first block is typically achievable. However, 2022 work by Anastasaki and coworkers overcame this limitation by employing a pH switchable RAFT agent and regulating its activity by addition of small aliquots of acid or base (Figure 19) [249]. This approach allowed for multiblock copolymer synthesis where the CTA activity could be tuned block‐by‐block. Multiblock copolymers with ascending, descending, alternating low and high dispersity values, or any combination thereof, could be targeted in a one‐pot synthesis by sequential monomer and acid/base addition in both aqueous and organic media [250]. Complex decablock copolymers with multiple dispersity variations were also synthesized to further demonstrate the potential of this methodology. More recently, An and coworkers demonstrated that the switchable RAFT agent approach to dispersity control was also effective in visible light photoiniferter RAFT [251], and can be used to tune the dispersity of star polymers by employing a tetrafunctional switchable RAFT agent [205].

**FIGURE 20:**
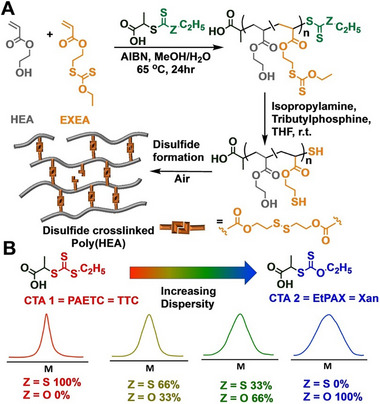
A) Synthesis of poly(HEA) disulfide crosslinked networks with mixed RAFT agents. B) Tuning the primary polymer dispersity using mixtures of trithiocarbonate (TTC) and xanthate (Xan) CTAs. Reproduced with permission [252]. Copyright 2024, John Wiley and Sons.

The ability of RAFT polymerization to precisely tune dispersity now opens the door to exploring its impact across a wide range of applications. For example, Konkolewicz and coworkers demonstrated that swelling ratio, tensile strength, and extensibility of polymer networks comprised of crosslinked linear chains were superior when the constituent chains had intermediate dispersity (*Đ* = 1.3‐1.5 for DP 100 and 1.6‐2.1 for DP 200) compared to materials with higher or lower dispersity (Figure 20) [252]. The degradation of polymer networks comprised of high dispersity crosslinked chains has also been shown to be up to 40% slower than for low dispersity analogs [253]. Kong et al. recently demonstrated that the mixed CTA strategy can be applied in photoenzymatic polymerizations to obtain ultra‐high molecular weight polymers with tunable dispersity [206]. Employing this strategy to form polymer networks revealed that the storage modulus scaled with MW, but peaked at intermediate dispersity values. In solution self‐assembly, Junkers and coworkers used RAFT to show that increasing dispersity of the core‐forming block reduces the overall dispersity of the nanoparticles. Furthermore, with the advent of techniques that enable dispersity control on a block‐by‐block basis, the self‐assembly properties of multiblock copolymers with tailored dispersities must be rigorously studied. This level of control over molecular weight distribution provides a new platform to investigate how dispersity affects both material properties and performance in advanced polymer systems.

### 4.6 Depolymerization

Radical polymerization has long been known to be an equilibrium process; a concept established in the 1940s. In this equilibrium, polymerization is generally favoured at lower temperatures, while depolymerization dominates at higher temperatures. The temperature at which these two processes are balanced is referred to as the ceiling temperature (*T*_c_). However, polymers synthesized through radical polymerization are typically kinetically trapped and will not automatically depolymerize when heated above the *T*_c_ of their constituent monomer [254]. To initiate depolymerization, a bond must first be broken to generate an active site, typically a radical species, that can facilitate depropagation. The thiocarbonylthio moieties present at the chain ends of RAFT polymers provide a versatile handle for reactivation, enabling further polymerization steps, such as the synthesis of block copolymers [255]. However, when activated at elevated temperatures, these moieties can also initiate depolymerization at significantly lower temperatures than conventional free radical polymerization (FRP) polymers [256].

The first depolymerization of a RAFT‐synthesized polymer was reported by Gramlich and coworkers in 2018 [257]. They showed that heating poly(poly(dimethylsiloxane) methacrylate) P(PDMSMA) and poly(oligo(ethylene glycol) methyl ether methacrylate) (POEGMA) bottle‐brush polymers could regenerate up to 35% monomer in 56 h, aided by the relatively low *T*_c_ of the monomers in question. In 2022, Anastasaki and coworkers reported the depolymerization of a wide range of poly(methacrylates) including poly(methyl methacrylate) (PMMA) [258]. By heating dilute polymer solutions in dioxane at 120°C, they demonstrated efficient depolymerization, regenerating up to 92% of the original monomer. Subsequent investigations found that depolymerization can occur from a range of different thiocarbonylthio end groups [259, 260], and activation of chains was facilitated by solvent‐derived radicals which undergo addition to the macroCTAs followed by fragmentation to yield polymeric radicals [261]. The end‐group unzipping nature of the depolymerization mechanism has since been confirmed by time‐resolved small‐angle X ray scattering, which showed that the weight‐average molecular weight gradually decreases during depolymerization, while the z‐average radius of gyration remains almost unchanged until ∼50% of the repeat units are converted [262]. Importantly, small molecule CTA‐derived species are recoverable post‐depolymerization, and these could be reused as effective RAFT agents in subsequent polymerizations, maintaining control over molecular weight and dispersity. A recent systematic study of methacrylates with varied side chains revealed that the rate of depolymerization is highly sensitive to the nature of the ultimate unit adjacent to the RAFT end group [263]. This suggests that activation of the polymer chain is the rate‐determining step in the depolymerization process. The depolymerizations demonstrated in these initial reports are relatively slow, typically reaching maximum conversion after around 8 h, likely due to the reliance on solvent‐derived radicals to activate the chain‐end groups. More efficient activation methods could improve both the speed and temperature conditions, enabling faster depolymerization, lower operating temperatures, and reducing the loss of end‐groups to side reactions [256]. For example, Mantzara et al. demonstrated that addition of convention free radical initiators allowed for much faster depolymerization in various solvents, and at higher repeat unit concentrations [264].

**FIGURE 21:**
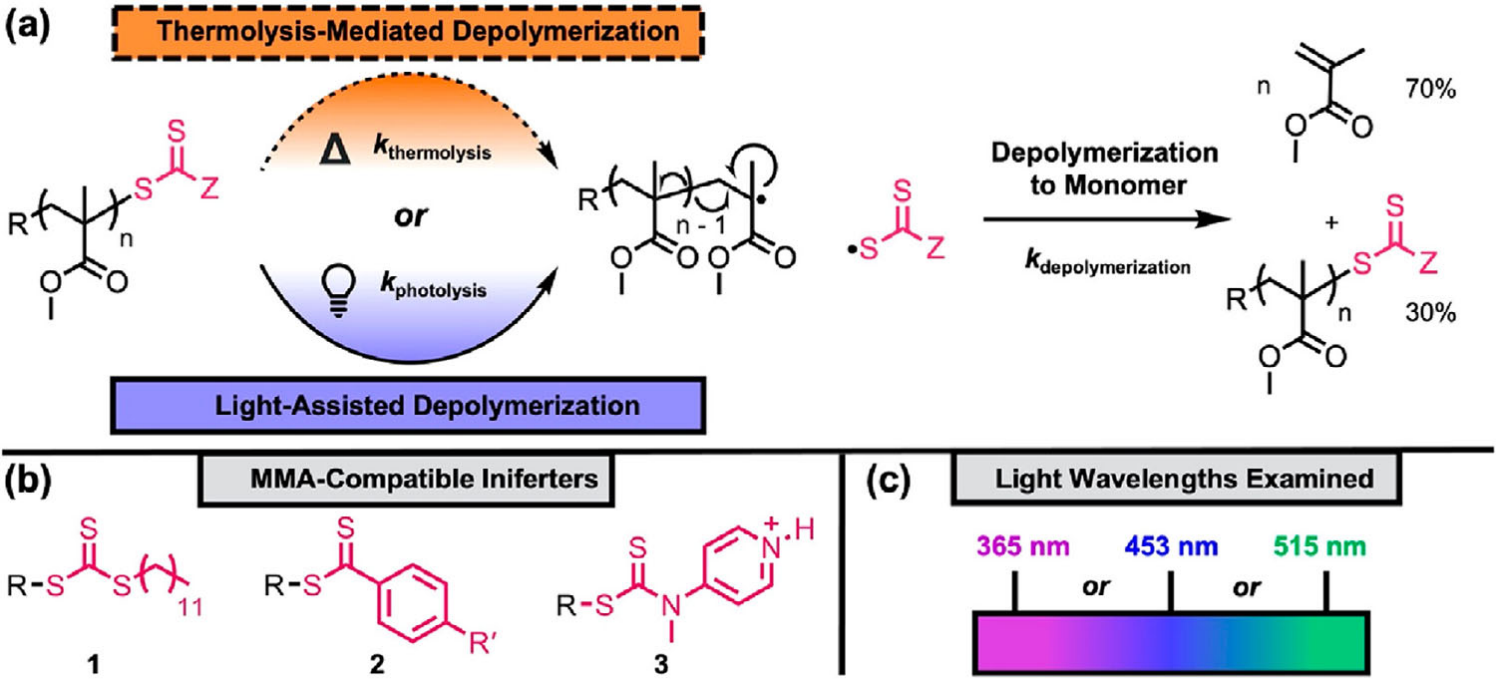
(a) Mechanistic pathways for thermolytic or photolytic‐initiated depolymerization of poly(methyl methacrylate) (PMMA). (b) Iniferters such as trithiocarbonates (1), dithiobenzoates (2), and dithiocarbamates (3) are capable of mediating the polymerization of more‐activated monomers such as methyl methacrylate (MMA). (c) Wavelengths of light used to excite electronic transitions in iniferters for depolymerization. Adapted with permission [265]. Copyright 2022, American Chemical Society.

To improve end‐group activation further, the groups of Sumerlin and Anastasaki independently introduced photomediated methods for chain‐end activation in the depolymerization of RAFT‐synthesized polymers. Sumerlin and coworkers employed a photoiniferter approach, leveraging n → π* and π → π* electronic transitions of the thiocarbonylthio end‐groups to promote depolymerization via direct bond homolysis (Figure 21). This method achieved up to 87% depolymerization within 1 h, and the depolymerization temperature was lowered to 100°C [265].

Anastasaki and coworkers utilized Eosin Y as a photocatalyst, similar to PET‐RAFT polymerization, to assist in activating the end groups at similarly reduced temperatures [266]. A subsequent study by Boyer and co‐workers further improved on these conditions by using a metalloporphyrin catalyst, which also introduced limited oxygen tolerance, though the process remained hindered by the low concentration of polymeric radicals [267]. Additionally, photocatalytic depolymerization strategies offer the possibility of temporal control over the reaction. Work by Bellotti et al. found that lower the depolymerization temperature to just 90°C minimized thermal initiation, meaning that photocatalytic RAFT depolymerization of poly(methacrylates) proceeds only in the presence of light, with no conversion observed during intermittent periods of darkness [268].

Boyer and coworkers demonstrated microwave‐assisted depolymerization of PMMA, poly(hydroxyethyl methacrylate), and poly(benzyl methacrylate) synthesized via RAFT polymerization [269]. The process exhibited strong temperature dependence, with substantial monomer recovery occurring between 110°C and 140°C, even at high repeat unit concentrations (up to 200 mM). Efficient depolymerization was achieved for polymers with molecular weights ranging from 5000 to 17 000 g/mol. Importantly, the RAFT end group played a critical role: polymers bearing dithiobenzoate end groups afforded the highest monomer recovery, whereas those with trithiocarbonate end groups yielded comparatively lower recovery.

A key drawback of the depolymerization approaches discussed so far is that, while they achieve high monomer conversion, there is little control over the process. Once polymer chains are activated, they fully unzip to monomer, as indicated by the minimal shift in molecular weight distribution (MWD) during depolymerization. This behaviour contrasts with RAFT polymerization, where chains grow concurrently in a controlled manner. In 2024, this limitation was addressed in the first report of controlled depolymerization of RAFT‐synthesized polymers [270]. Control over the depropagation could be attained by either adding a highly active RAFT agent to the depolymerization or by increasing the polymer concentration so that the CTA concentration was sufficiently high to deactivate polymer chains during the process. By doing so, a gradual decrease in the polymer's *M*_n_ was observed as depolymerization proceeded. This advancement allowed depolymerization to be used as an analytical technique for sequencing block copolymers. For example, AB and BA type block copolymers were successfully depolymerized in a controlled manner, with the monomer adjacent to the end‐group appearing first in the depolymerization products, followed by the second monomer. A statistical copolymer of monomers A and B showed both monomers appearing simultaneously in the products. A related advancement by Sumerlin and coworkers demonstrated that uncontrolled RAFT depolymerization can also be applied selectively to narrow molecular weight distributions [271]. In a mixture of polymers prepared by free radical polymerization and RAFT polymerization, only the RAFT‐derived chains were capable of depolymerizing under thermal conditions. By tuning the depolymerization time and conditions, shorter RAFT chains were selectively depolymerized while longer chains remained intact, effectively “sculpting” the overall MWD. This approach establishes depolymerization as a tool not only for monomer recovery or structural analysis, but also for post‐polymerization refinement of polymer blends.

Depolymerizations are typically conducted in solution, with dilution favouring the shift in equilibrium toward monomer regeneration. However, a bulk depolymerization approach could offer significant advantages. Whitfield et al. recently demonstrated solvent‐free depolymerization of RAFT polymers at 200°C, providing evidence that partial macromonomer formation is a key factor behind the high depolymerization efficiencies observed [272]. This approach minimizes high‐temperature scission events and side reactions, thereby reducing monomer waste and enabling the recovery of high amounts of pristine monomer on scales up to 10 grams. In parallel, Sumerlin and coworkers developed a bulk depolymerization process for poly(methyl methacrylate) (PMMA) at significantly lower temperatures than conventional methods, by incorporating thermolytically labile end‐groups [273]. The combination of α‐end *N*‐hydroxyphthalimide esters and ω‐end trithiocarbonates allowed for over 90% MMA recovery.

The depolymerization of RAFT‐synthesized polymers is an exciting and emerging area of research, but it has so far been largely limited to polymethacrylates. Recently, the scope of RAFT depolymerization was expanded to include polymethacrylamides, as demonstrated by Lohmann et al. [274]. By optimizing reaction conditions and introducing a free radical initiator, depolymerization was achieved at temperatures as low as 90°C with >70% monomer regeneration, demonstrating that high depolymerization efficiency is attainable even for challenging polar monomers. Expanding RAFT depolymerization to other monomer classes remains a significant challenge that must be addressed for broader applicability. Another major limitation is the reduced depolymerization efficiency observed at high molecular weights, which limits scalability and practical application. To address this, Boyer and coworkers developed a strategy that retroactively installs RAFT functionality into polymers originally synthesized by free radical polymerization [275]. High molecular weight polymethacrylates were selectively cleaved using hydrogen atom transfer (HAT) chemistry [276] to generate new chain ends, which were then functionalized in situ with RAFT agents. These modified polymers underwent efficient depolymerization, enabling monomer regeneration from multiple points along the chain. Up to 53% monomer recovery was achieved within just 5 h, representing a major advance toward the depolymerization of commodity polymers aided by RAFT chemistry.

### 4.7 3D Printing

**FIGURE 22:**
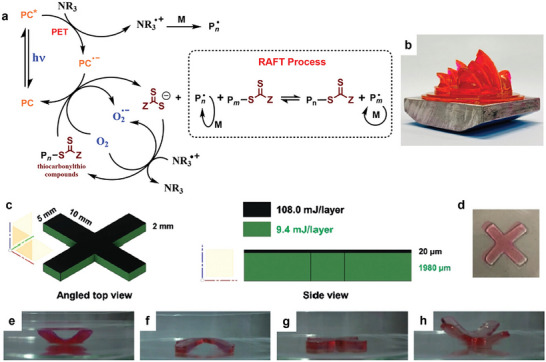
Fabrication of 3D/4D printed materials by PET‐RAFT polymerization. a) Proposed PET‐RAFT mechanism using a photocatalyst and tertiary amine co‐catalyst. PC: photocatalyst; NR_3_: tertiary amine; PET: photoinduced electron transfer. Adapted with permission [277]. Copyright 2019, John Wiley and Sons. b) 3D printed materials via PET‐RAFT polymerization. Adapted with permission [278]. Copyright 2021, American Chemical Society. c,d) Spatially differentiated irradiation doses provide 4D printed materials that displayed e–h) solvent induced actuation. Adapted with permission [277]. Copyright 2019, John Wiley and Sons.

3D printing is an additive manufacturing technology that fabricates objects with high shape complexity in a layer‐by‐layer manner based on digitally sliced computer‐aided designs. Since its inception in the early 1980s, various 3D printing techniques have been developed. Among these, vat photopolymerization methods such as digital light processing (DLP) and stereolithography (SLA) are widely used. These techniques leverage light to selectively cure liquid resins in a vat, forming solid materials. Historically, DLP and SLA relied on uncontrolled photopolymerization methods, such as conventional free‐radical or cationic photopolymerizations. While these methods enable rapid polymerization, which is ideal for constructing 3D objects efficiently, they offer minimal control over polymer chain growth at the macromolecular level. Due to recent advancements in polymer chemistry, particularly the development of oxygen‐tolerant photoRAFT polymerization (e.g., photoinitiated RAFT and PET‐RAFT polymerization), these techniques have been implemented in vat photopolymerization by enabling precise control over polymer network formation. Bagheri and Boyer were among the first to demonstrate the integration of PET‐RAFT polymerization into 3D stereolithography [277]. In their pioneering work, erythrosine and EY were used as PCs to mediate reductive PET‐RAFT polymerization under green and blue light (450 nm and 530 nm), facilitating the fabrication of 3D‐ and 4D‐ printed materials (Figure 22). For example, 3D printing using PET‐RAFT polymerization was achieved by formulating resins that combined erythrosine, TEtOHA, *N,N*‐dimethylacrylamide (DMAm), and poly(ethylene glycol) diacrylate (PEGDA, *M*_n_ = 250 g/mol). Optimization of reagent stoichiometry allowed rapid polymerization, with a build speed of up to 1.2 cm/h, facilitating the successful fabrication of complex structures (Figure 18). Furthermore, PET‐RAFT polymerization has been employed to fabricate 4D printed materials that exhibit swelling and desolvation‐induced actuation (Figure 18). The choice of RAFT agents—including trithiocarbonates (TTCs), dithioesters, xanthates, and dithiocarbamates—has been shown to significantly influence polymerization kinetics and must be carefully considered during the formulation process for 3D printing applications [278]. In a related study, Bagheri et al., in collaboration with Boyer's group, developed a PET‐RAFT polymerization system employing EY as a PC and triethylamine as a reducing agent for 3D printing using blue or green light [279, 280]. However, the fastest achievable build speeds were 0.08 and 0.14 cm/h under blue and green light irradiation, respectively, which is lower compared to systems using erythrosine. However, the presence of PCs can be disadvantageous in some specific applications, to overcome this challenge, PI using first trithiocarbonate, was also attempted for 3D printing, although the building speed was extremely low (0.01–0.05 cm/h) [281]. Zhu reported a fast building speed when xanthate was employed as RAFT agent, which was attributed to its lower chain transfer constant [282].

Incorporating RAFT polymerization not only provides control over polymer network formation but also enhances the mechanical properties (e.g., toughness) of printed materials compared to those produced using conventional photopolymerization [283]. Furthermore, this approach enables post‐functionalization of 3D‐printed objects, which is not possible with traditional methods, enabling the modification of the surface of 3D printed materials.

However, a significant limitation of early PET‐RAFT 3D printing technique was its slower printing speed compared to commercial 3D printing methods (build speed around 1.2 cm/h), depending on the PC used [284]. To address this issue, Boyer's group further explored photoinitiated RAFT polymerization strategies, aiming to optimize the process for improved efficiency without compromising the benefits of controlled polymerization [285]. In one notable study, they demonstrated by optimizing the ratio RAFT ((n‐butylthiocarbonothioylthio) propanoic acid (BTPA)): photoinitiator (diphenyl (2,4,6‐trimethylbenzoyl) phosphine oxide (TPO)) the rapid fabrication of 3D printed objects with building speed up to 9.1 cm/h. Interestingly, the RAFT agent not only enabled controlled polymerization but also improved the resolution of 3D printing. By absorbing light, the RAFT agent minimized overcuring, allowing for more precise control over feature dimensions. Additionally, the incorporation of RAFT agents endowed the printed materials with advanced properties, such as self‐healing and post‐functionalization capabilities. For instance, Zhang et al. demonstrated the ability of RAFT agents within the polymer network to facilitate self‐healing in damaged 3D‐printed objects. By adding small amounts of monomers and crosslinkers to the damaged area and exposing the samples to 360 nm UV light, the RAFT agent enabled chain extension and reformation of the polymeric network, effectively repairing the material [286]. In another example, Lee et al. leveraged the presence of RAFT agents on the surface of 3D‐printed materials to grow various polymer chains, allowing for the modification of surface properties, tuning the hydrophobicity, for instance [285]. This post‐functionalization capability provided a versatile approach to tailor the surface characteristics of 3D‐printed objects for specific applications.

The ability to chain‐extend polymers prepared using RAFT agents has also been exploited for the fabrication of 3D printed nanostructured materials with distinct domains exhibiting different properties. This approach utilizes polymerization‐induced microphase separation (PIMS) [287, 288, 289], where the controlled growth of polymer chains leads to the formation of nanoscale domains with tailored compositions and functionalities during the 3D printing process [290, 291]. In this method, instead of adding a RAFT agent directly to the resin, a macro‐RAFT agent is dissolved into the resin, containing monomer, crosslinker, and photoinitiator. This macro‐RAFT agent is then chain‐extended with monomers and crosslinkers, leading to the formation of block copolymers, resulting in phase separation. One resulting domain remains crosslinked, providing structural stability, while the other remains non‐crosslinked, offering flexibility and functionality. The first reported system for 3D printing used poly(n‐butyl acrylate) (PBA) as macro‐RAFT agent, acrylic acid (AA) and polyethylene glycol diacrylate (PEGDA) as chain extension monomers/crosslinkers. The materials formed through this technique typically display globular, elongated globular, and co‐continuous morphologies. In the case of co‐continuous morphology, each domain is fully connected (continuous) throughout the material, and the domains are locally, but not globally, ordered. The co‐continuous morphologies formed via this process are arguably the most relevant nanostructures from a practical materials perspective as these materials have been used in diverse applications, including drug delivery [292], energy storage (solid polymer electrolyte) [293, 294, 295, 296], and many others [296, 297]. The formation of nanodomains can be precisely controlled by adjusting the chain length of the macro‐RAFT agent incorporated into the resin [284, 290, 291, 298], as well as by varying its overall content [291]. The characteristic domain size is strongly influenced by the radius of gyration of the diblock copolymer formed during chain extension, offering a straightforward method to regulate nanodomain formation [298]. In addition to this, other strategies have been reported to tune the size and morphology of nanodomains. The reactivity of the RAFT end group in the macro‐RAFT agent significantly affects chain extension and, consequently, nanodomain formation [299]. Furthermore, the inclusion of non‐reactive homopolymers alongside the macro‐RAFT agent in the resin formulation can impact domain structure. Additionally, the architecture of the macro‐RAFT agent plays a critical role in determining domain morphology [300, 301, 302]. By altering the macro‐RAFT architecture from a linear structure to 3‐arm or 4‐arm star configurations, the domain morphology can be transformed from bicontinuous structures to inverted morphologies [300]. The morphology of nanodomains plays a pivotal role in defining the properties of these materials [290, 291]. For example, nanostructured 3D‐printed materials with interpenetrating soft and hard domains, particularly those exhibiting elongated or bicontinuous morphologies, demonstrate superior mechanical performance compared to materials with isolated globular domains [290]. This enhancement is primarily due to the increased interfacial interactions between the soft and hard domains, which promote efficient stress distribution and alleviate stress concentrations throughout the material. In addition to mechanical properties, the morphology of nanodomains also significantly influences swelling behaviour, enabling the design and fabrication of advanced 4D materials with dynamic, responsive properties [300].

### 4.8 SUMI/Step‐growth RAFT

RAFT Single Monomer Unit Insertion (SUMI) is a process where a single monomer unit is incorporated into RAFT agents (degree of polymerization, DP  =  1). Historically, thiocarbonylthio compounds have been employed for SUMI to design complex molecules [303, 304, 305]. However, with the advent of RAFT polymerization, RAFT‐SUMI has emerged due to its high efficiency [306, 307]. Innovations in photo‐mediated RAFT techniques, such as photoinduced electron transfer RAFT (PET‐RAFT), have enabled finer sequence control with increased selectivity and reduced side reactions [308]. Significant advancements have been achieved in the recent decade with iterative SUMI using PET‐RAFT to design sequence‐defined molecules initially demonstrated by Boyer's group [309, 310, 311].

Recently, Xu et al. expanded the library of possible sequences by iterative SUMI, alternating between electron‐rich and electron‐poor monomers with low propagation rate constants (k_p_) [312]. By employing solid‐phase support, they achieved 18 iterative insertions composed of indene‐maleimide units [313]. Although the yield was 2%, this was unprecedented for RAFT‐SUMI, showcasing a potential strategy for preparing sequence‐defined polymers.

Furthermore, Xu et al. highlighted that the penultimate monomer in the sequence affects the rate and degradation with PET‐RAFT SUMI [314, 315]. In a recent study, they found that changing the Z‐group can affect the overall yield in some cases [316]. Kinetic studies have shown that PET‐RAFT SUMI follows a rate order of 1.5 with respect to the RAFT agent, highlighting the dependence on TTC for photo‐activation [314, 317]. This contrasts with the pseudo‐first‐order kinetics typically observed in thermally initiated RAFT‐SUMI processes using azo‐initiators [318].

**FIGURE 23:**
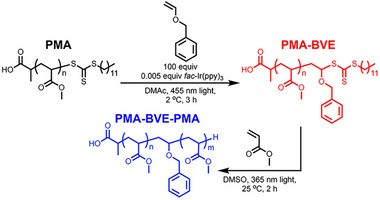
Scheme of the insertion of BVE into a PMA chain and subsequent chain extensions from poly(methyl acrylate)‐benzyl vinyl ether (PMA‐BVE) using methyl acrylate to achieve PMA‐BVE‐PMA. Adapted with permission [319]. Copyright 2023, American Chemical Society.

Another unique utility was reported by Figg, who employed PET‐RAFT SUMI on the RAFT polymer end‐group, followed by chain extension to install a single monomer unit in the middle of an acrylic polymer (Figure 23) [319]. This novel application of RAFT‐SUMI highlights the potential for incorporating single functionalities along a polymer chain.

An exciting innovation was observed by employing RAFT SUMI for step‐growth polymerization [320], using bifunctional monomers and RAFT agents tethered by the R‐group. This forms a polymer backbone with TTC pendant side chains, allowing the preparation of graft copolymers with unique functional step‐growth backbones for stimuli‐responsive degradation [321]. This unique polymerization was demonstrated with conjugated monomers such as maleimide [320, 322, 323], acrylic [324, 325], acrylamide [326], and styrenic [327] monomers by employing trithiocarbonate‐based TTC, as well as with non‐conjugated monomers such as vinyl ethers [328], vinyl esters [329], and allylic monomers [330] using xanthate‐based TTC. This approach has been used to generate fluorinated polymers for coatings [331], as well as modifiable and degradable polymer networks [330]. Additionally, 3D printing using this approach with a tetrafunctional RAFT agent was reported [329], yielding materials with a more tunable Young's modulus.

Additionally, RAFT step‐growth polymerization via the Z‐group approach was recently reported, where the polymer backbone is tethered via the Z‐group by employing a symmetric trithiocarbonate that bears two R‐groups [327]. In contrast to the former approach, a “dynamic” main chain is formed, allowing partial depolymerization of the polymer backbone through RAFT metathesis processes. Moreover, embedded TTC units in the backbone allow main‐chain expansion when subjected to RAFT chain‐growth conditions, enabling facile synthesis of deconstructable multiblock copolymers [327].

**FIGURE 24:**
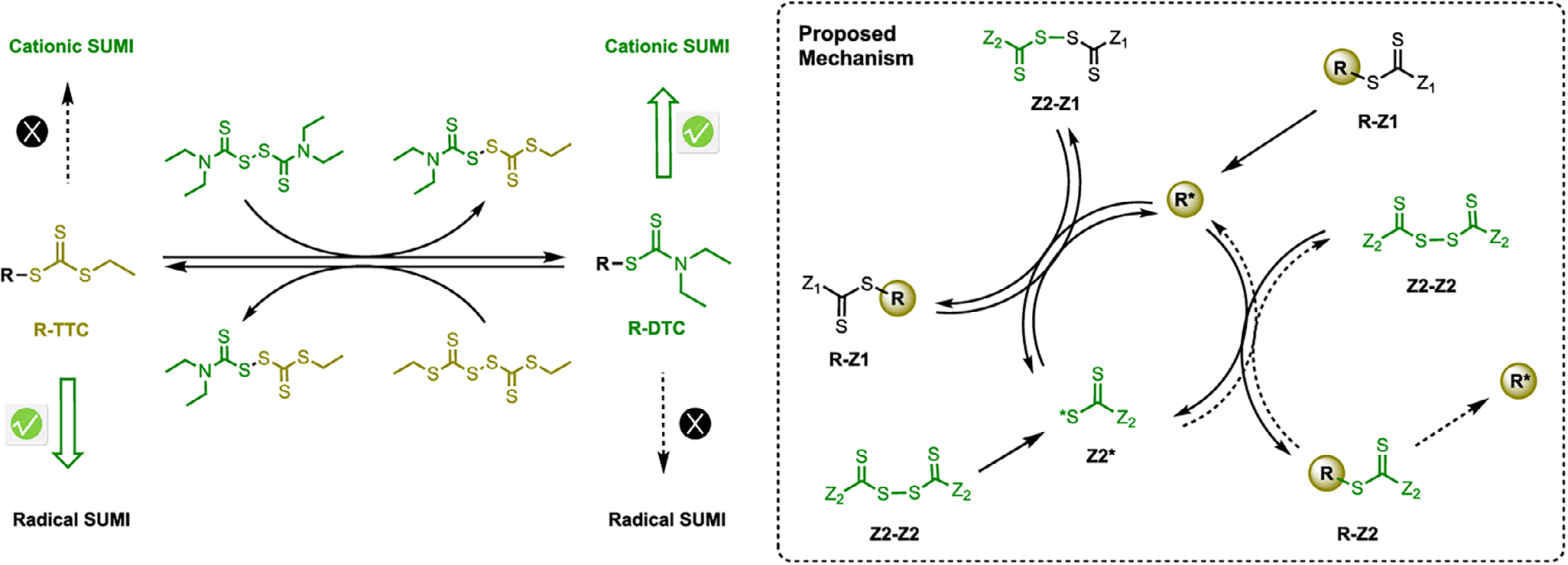
Interconvertible radical and cationic SUMIs by switching thiocarbonylthio end‐groups between trithiocarbonates to dithiocarbamates, and proposed mechanism for thiocarbonylthio exchange reaction between chain transfer agents and bis(thiocarbonyl) disulfides. Reproduced with permission [332]. Copyright 2024, Springer Nature.

Recently, Liu and Yang demonstrated tandem radical and cationic RAFT SUMI by switching the TTC end group through disulfide exchange to interchange between trithiocarbonate and dithiocarbamate to increase the efficiency of radical and cationic RAFT exchange, respectively (Figure 24) [332]. Subsequently, the same group demonstrated concurrent orthogonal cationic and radical SUMI‐mediated step‐growth polymerization [119].

A fundamental parameter that increases efficiency in the RAFT‐SUMI process was disclosed by Tanaka to be a high ratio of the forward to reverse chain transfer constants (C_tr_⁄C_–tr_) [333], aptly described as the SUMI efficiency ratio. A higher C_tr_⁄C_–tr_ indicates a more favorable formation of DP  =  1 over DP  =  2 under rapid RAFT exchange conditions; thus, optimizing this ratio leads to increased selectivity for SUMI and yield.

## 5 Future Outlook

In the past five years since our last review, we have once again witnessed the rapid development of RAFT polymerization across various aspects, including initiation methods, reaction environmental conditions, and expanded applications.

For photoiniferter RAFT (PI‐RAFT), there have been significant improvements in reaction rates achieved either by using stronger irradiation sources or by employing special solvents such as deep eutectic solvents or ionic liquids. These polar solvents not only stabilize the RAFT agent but also significantly enhance reaction rates—by up to fivefold in some cases. Moreover, oxygen tolerance has been observed when using ionic liquids. Future studies will likely further explore the role of oxygen in PI‐RAFT processes.

In the case of photo‐electron transfer RAFT (PET‐RAFT), we have seen a growing variety of photocatalysts, particularly those capable of absorbing long‐wavelength light that can penetrate solid or turbid media. The role of reducing agents in PET‐RAFT is also gaining increasing attention. We anticipate further development in this area, including systems that combine multiple photocatalysts in a cascade or tandem fashion.

RAFT polymerization can also be temporally controlled using external energy sources such as electricity (eRAFT) and ultrasound (Sono‐RAFT). Both methods enable “on‐off” switching of polymerization. eRAFT has now been successfully applied in emulsion systems, while Sono‐RAFT has progressed into flow reactor systems. However, oxygen sensitivity remains a challenge for both techniques.

Cationic RAFT has facilitated stereoselective polymerization and enabled the polymerization of monomers that are typically incompatible with conventional RAFT. We've seen a broader range of catalysts and monomers explored. Notably, this method is increasingly being combined with other polymerization techniques, which expands its versatility. Very recently, control of anionic polymerization using RAFT and DT mechanisms has also been reported [334, 335, 336]. The use of the RAFT mechanism in various polymerizations is further expected.

Fenton‐RAFT has emerged as a fast and scalable option. It uses naturally occurring Fenton reagents—hydrogen peroxide and ferric ions—allowing polymerization to proceed in biological environments such as cellular media. This method also enables modulation of hydroxyl radical generation, thus offering control over chain‐end fidelity. One standout feature of Fenton‐RAFT is its ability to produce ultra‐high molecular weight polymers. When heterogeneous Fenton catalysts are employed, their introduction and removal can provide temporal control over the reaction. Photo‐Fenton RAFT, which uses light to promote hydroxyl radical formation, has also been reported and offers a direct method for temporal control. There is increasing interest in using enzyme‐assisted and broad‐spectrum photocatalytic systems to control hydroxyl radical generation.

We have also observed progress in mechano‐redox RAFT, where mechanical forces or low‐frequency ultrasound induce radical formation to initiate polymerization. In addition, acid‐triggered RAFT has emerged as a novel method, expanding the toolbox for activating RAFT processes.

Recent studies have explored conducting RAFT polymerization in special environments, including within bacterial and cancer cells. These developments pave the way for exciting new biological applications, and we expect this area to grow significantly in the coming decade.

Oxygen tolerance remains a critical area of focus due to its potential to simplify scaling. Oxygen may be removed by physical or chemical methods prior to polymerization or incorporated into the initiation mechanism—as in bio‐Fenton RAFT. Understanding the precise role of oxygen in RAFT systems remains an important direction for future research and could benefit from new solvents or photocatalysts.

Alternative RAFT‐like chain transfer agents, such as selenium‐based agents, have been developed in parallel. Producing ultra‐high‐molecular‐weight RAFT polymers has long been a field goal, and various strategies—such as improved trithiocarbonate (TTC) agents, the gel polymerization concept, and mini‐emulsion polymerization techniques—have shown success in achieving molecular weights over 1 MDa with high chain‐end fidelity.

Polydispersity control and depolymerization have also emerged as important topics. Recent studies show that chain‐end fidelity does not necessarily correlate with polydispersity. Using pH‐switchable RAFT agents, it is now possible to tune dispersity during block extension. Methods for on‐demand depolymerization by reactivating RAFT end groups have improved, offering a new avenue for polymer lifecycle control. The ability to depolymerize at the end of a material's functional life is particularly promising, as is applying the principles of RAFT depolymerization to polymers synthesized by free radical polymerization.

The range of RAFT applications continues to expand. A major area of growth is 3D printing using PET‐RAFT, where printing speed has historically been a limitation. New catalyst designs have led to faster polymerization, and improving speed remains a priority. Another innovative technique is RAFT Single Unit Monomer Insertion (SUMI), which allows for the creation of sequence‐defined polymers using PET‐RAFT. SUMI also enables hybrid processes combining step‐growth polymerization with single‐monomer functional insertions, opening the door to sophisticated polymer architectures with controlled degradability.

Lastly, RAFT polymerization in flow reactors has gained momentum. Flow systems facilitate robotic synthesis, enabling large‐scale data collection for machine learning‐based optimization and discovery. In the past five years, we've seen examples of flow RAFT polymerizations integrated with AI for targeting and molecular weight precision. We have also seen the robotic RAFT polymerization in a batch reaction system. We expect rapid advances in this field, enabling on‐demand synthesis of polymers with customized chemical and physical properties.

## Conflicts of Interest

The authors declare no conflicts of interest.

## Data Availability

The authors have nothing to report.
